# MultiCook: A Tool That Improves Accuracy of HLA Imputation by Combining Probabilities From Multiple Reference Panels and Methods

**DOI:** 10.1111/tan.70153

**Published:** 2025-05-06

**Authors:** Hakin Kim, Hyunjoon Lim, Buhm Han

**Affiliations:** ^1^ Interdisciplinary Program for Bioengineering Seoul National University Seoul Republic of Korea; ^2^ Convergence Research Center for Dementia Seoul National University Medical Research Center Seoul Republic of Korea; ^3^ Department of Biomedical Sciences BK21 Plus Biomedical Science Project, Seoul National University College of Medicine Seoul Republic of Korea

**Keywords:** diverse ethnicity, HLA imputation, reference panel

## Abstract

HLA molecules are produced by genes within the Major Histocompatibility Complex (MHC). Although the identification of HLA genotype is costly, fortunately, recent computational methods have made it possible to impute HLA genotypes using inexpensive single nucleotide polymorphism (SNP) markers. These imputation methods perform well if ethnicity‐matched reference panels are provided. However, the availability of large‐sized panels specific to each ethnicity remains limited. As a result, to achieve better imputation for each population, we need to utilise available multiple reference panels together. In this study, we introduce MultiCook, which enables users to simultaneously utilise existing multiple HLA imputation methods with multiple reference panels. MultiCook is versatile in that panels typed with different SNP genotyping platforms can seamlessly be merged, outputs from multiple imputation methods can be combined and the output from the Michigan imputation server with a multiethnic reference panel can also be incorporated. We compared MultiCook to the existing single‐reference‐panel approaches and the Michigan HLA imputation server. In evaluation with a cohort of 413 Koreans, MultiCook reduced the imputation error rate by about one third, from 4.70% to 3.31%, by combining 1KG EAS (*N* = 504) and HAN Chinese (*N* = 9773) reference panels compared to the single‐panel approach. Moreover, MultiCook achieved better accuracy for imputing low‐frequency alleles in evaluation benchmarks.

## 1 Introduction

HLA molecules are produced by genes within the Major Histocompatibility Complex (MHC) on Chromosome 6. They play a key role in recognising self and non‐self, responding to antigenic stimulation and regulating both cellular and humoral immunity [1]. In transplantation, HLA matching is vital since different HLA genotypes can lead to rejection [2]. The DNA sequence variation of HLA is closely associated with various human immune diseases, making fine‐mapping analysis essential to identify specific HLA alleles critical for disease development [3]. Fine‐mapping analysis requires a large number of samples due to the complex genetic structure, polymorphism and extensive linkage disequilibrium in the MHC region.

For HLA typing, NGS, SSOP and SBT are commonly used. NGS, based on targeted sequencing and allele phasing, provides detailed HLA alleles with up to four fields of resolution [4]. In contrast, SSOP uses sequence‐specific hybridisation and SBT relies on Sanger sequencing, both typically offering two fields of resolution [4]. These methods are more expensive than genome‐wide single‐nucleotide polymorphism (SNP) arrays. Consequently, obtaining HLA genotype data for large samples is challenging due to the cost involved [5, 6].

While SNP‐based HLA imputation is not as concordant with true HLA genotypes as conventional HLA typing technologies, SNP‐based HLA imputation, which predicts HLA genotypes using SNP data, is cheaper, making it useful for population studies and genetic epidemiology. Genome‐wide SNP arrays cost less than half of the aforementioned technologies [6]. Consequently, several HLA imputation methods have been developed to predict HLA genotype data from SNP data [7, 8, 9, 10]. One such method, SNP2HLA, developed by Jia et al. [5], translates multiallelic HLA information into binary markers so that an imputation engine (Beagle v3) [11] can be applied to perform HLA imputation. CookHLA, an extension of SNP2HLA by Cook et al. [8], employs a more advanced engine (Beagle v4 and v5) and improves accuracy by embedding prediction markers locally within each polymorphic exon. Zheng et al. developed HIBAG, an imputation method based on attribute bagging [10]. These classical methods rely on a single reference panel for HLA imputation. Recently, Luo et al. [12] implemented an HLA imputation pipeline on the Michigan server, which offers a user‐friendly imputation with a multi‐ethnic HLA reference panel consisting of 20,349 samples.

The accuracy of HLA imputation is affected by various properties of reference panels, including the size, the number of shared SNPs with the target data and the ethnic similarity between the panel and the target data [13, 14]. Additionally, the HLA typing method and the level of ethnic diversity within the reference panel play key roles [14]. Therefore, selecting an optimal reference panel is essential for accurate imputation [9, 15]. Generally, choosing the largest panel among ethnicity‐matching reference panels is recommended. However, large‐sized, ethnicity‐matching HLA reference panels are limited, especially for underrepresented populations. Moreover, the coverage of HLA alleles and SNPs by a single reference panel is constrained.

Employing multiple reference panels can effectively overcome these limitations. Combining multiple panels increases the reference sample size and expands the coverage of HLA alleles and SNPs. Additionally, it enhances diversity, particularly, for samples from countries lacking specific HLA panels, by maximising diversity within the broad Asian ethnicity. One effort for utilising multiple reference panels is MergeReference [16], which constructs a merged reference panel by identifying overlapping SNPs and HLA genes from available reference panels. However, this method has limitations, as different genotyping technologies can restrict the number of overlapping SNPs. Combining multiple panels results in a small number of SNPs common to all panels. Another effort is building multiethnic HLA reference panels created using multiple panels from NGS by international collaboration [6, 12, 17]. Multiethnic HLA reference panels can improve the imputation of rare alleles in underrepresented populations [17], but may still lack sufficient ethnicity‐matching samples for a better imputation performance, which requires ethnic similarity between the panel and the target data. Furthermore, these multiethnic HLA reference panels are only accessible through outsourced imputation services that employ predetermined HLA imputation tools such as SNP2HLA or HIBAG within their established pipelines, rather than being directly available.

In this paper, we introduce MultiCook, which combines multiple results of CookHLA [8] based on a single ethnicity‐matching reference panel. MultiCook can also merge the results of the Michigan imputation server [12] and HIBAG [10]. Instead of directly merging reference panels before imputation, MultiCook combines imputation probabilities sequentially after applying existing HLA imputation methods. Specifically, for each two‐field HLA allele, MultiCook extracts the imputation probability from each imputation result and merges the probabilities of multiple results into a single value. MultiCook offers three key advantages. First, it enhances imputation accuracy by increasing the number of reference panels. For example, when imputing HLA for Koreans, combining the HAN Chinese [18] panel with the 1KG EAS [12] panel reduced the imputation error from 4.70% to 3.31%, a reduction of about one‐third compared to using a single panel (CookHLA based on 1KG EAS). Second, MultiCook improves imputation accuracy for rare alleles. Single reference panels often miss specific low‐frequency alleles due to limited sample sizes, resulting in a zero probability of imputing those alleles. By utilising multiple panels, MultiCook can potentially predict all genes and alleles included in the union of the panels, thereby improving rare allele prediction, as demonstrated in our benchmark. Third, MultiCook is flexible in incorporating results from both CookHLA [7] and external resources like the Michigan HLA imputation server [9], which uses a built‐in large‐sized multi‐ethnic HLA reference panel. By using an ethnicity‐matching reference panel as well as a multiethnic panel, MultiCook can increase the ethnic similarity between the reference panel and the target data. MultiCook is freely accessible at https://github.com/2w21234/MultiCook/. We provide a local web‐based platform for MultiCook, broadening accessibility. The interface allows users to run single‐reference‐panel imputation methods (CookHLA and HIBAG) and merge outputs of the Michigan Imputation Server. We attached the manual for local web‐based MultiCook in Data S1.

## 2 Methods and Materials

### 2.1 Imputation Procedure of MultiCook

**FIGURE 1:**
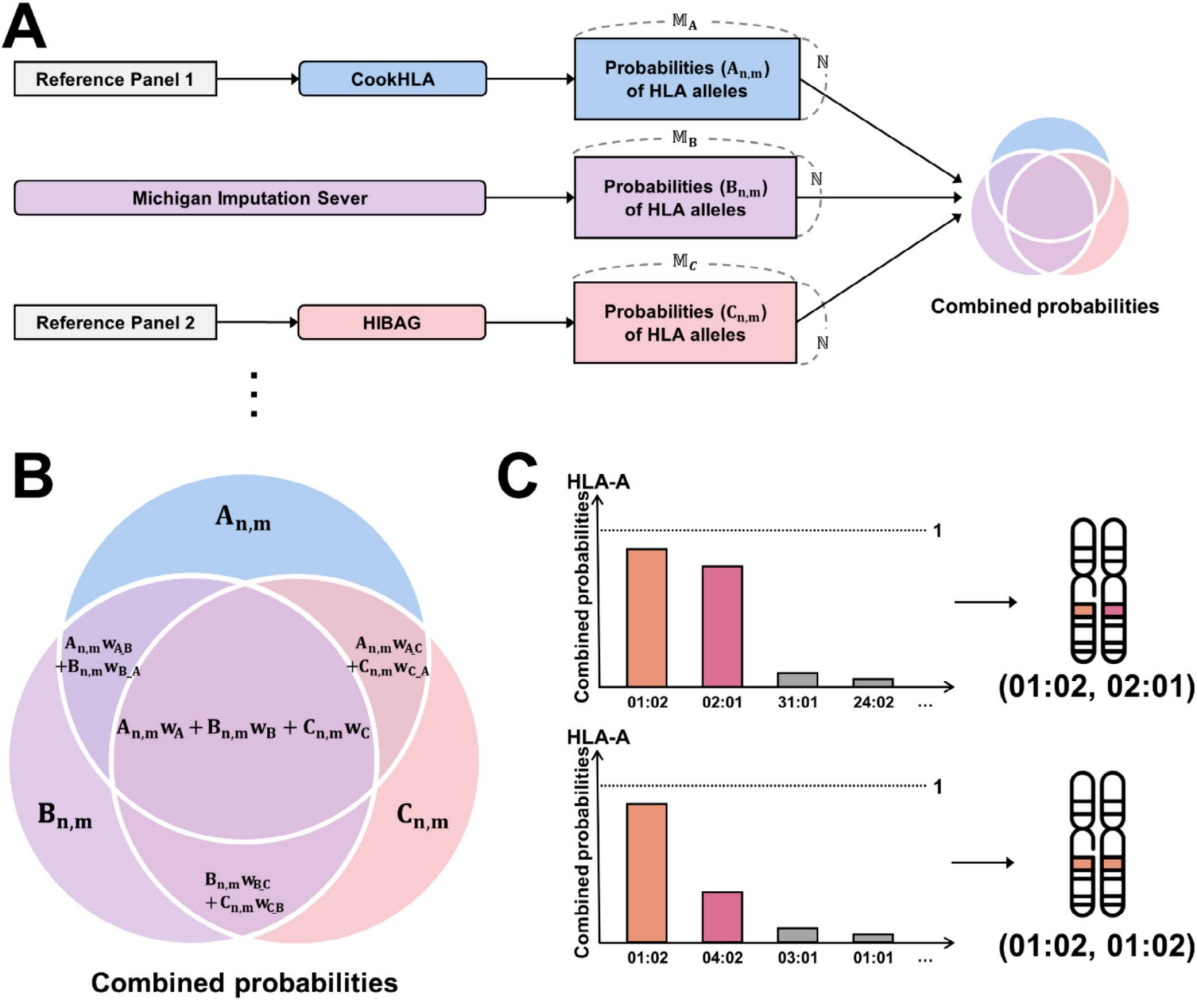
Overview of MultiCook. MultiCook integrates HLA imputation results from single‐panel‐based methods (CookHLA, the Michigan imputation server and HIBAG). The process consists of three steps: (A) MultiCook first obtains posterior probabilities for each HLA allele from the single‐panel methods. (B) Next, it linearly combines these probabilities using user‐specified weights. (C) Finally, MultiCook calls HLA genotypes based on the combined probabilities. For the two alleles with the highest combined probabilities, MultiCook assigns a homozygous genotype if the highest probability is more than twice that of the second‐highest. Otherwise, it assigns a heterozygous genotype.

MultiCook merges imputation results from CookHLA, HIBAG and the Michigan imputation server, each based on independent and diverse reference panels. It assumes that two‐field HLA alleles are imputed using these methods before sequentially merging the results. To simplify the workflow, MultiCook provides scripts for running CookHLA and HIBAG before the merging process, ensuring user convenience. In the merging step, MultiCook operates through three steps (Figure 1). Although the figure demonstrates combining three imputation results, MultiCook allows for merging any number of imputation results.

**Step 1**. Calculating the posterior probability of the allele's presence.

For each two‐field HLA allele, we calculate the posterior probability of the allele presence. The posterior probability is calculated using the HLA genotype probabilities, which consist of reference homozygous, heterozygous and alternative homozygous (Figure 1A). The reference homozygous genotype is denoted as *Presence/Presence*. The heterozygous genotype can be either *Presence/Absence* or *Absence/Presence*. The alternative homozygous genotype is represented as *Absence/Absence*. We define the posterior probability as:

(1) $$\text{Posterior probability}=\hat{\mathrm{RH}}+\frac{\hat{H}}{2}$$

where $\hat{\mathrm{RH}}$ is the estimated genotype probability of reference homozygous and $\hat{H}$ is the estimated genotype probability of the heterozygous. For example, assume that an individual has genotype probability values of 0.68, 0.32 and 0 for the reference homozygous, heterozygous and alternative homozygous genotypes for HLA‐A*02:01. The posterior probability that the person has HLA‐A*02:01 is calculated to 0.68 + 0.32/2 = 0.84.

**Step 2**. Combining posterior probabilities.

For each two‐field HLA allele of an individual, the posterior probabilities from multiple reference panels are linearly combined at specified weights to calculate the merged posterior probability (Figure 1B). Let $A_{n,m}$, $B_{n,m}$ and $C_{n,m}$ denote nth individual's mth allele's posterior probabilities from the reference panels A,B and C. These posterior probabilities are obtained using equation (1). Let $M_{A}$, $M_{B}$ and $M_{C}$ represent the sets of two‐field HLA allele markers present in each reference panel. Here, the weights for A, B and C are denoted as $w_{A}$, $w_{B}$ and $w_{C}$, respectively. The weights are specified by the user to reflect the expected contribution of each reference. Then, the merged posterior probability of the nth individual is

(2) $$P_{n,m}=\left\{\begin{array}{cc} \begin{array}{c} \begin{array}{c} A_{n,m}w_{A}+B_{n,m}w_{B}+C_{n,m}w_{C}\\ A_{n,m}w_{A\_B}+B_{n,m}w_{B\_A} \end{array}\\ A_{n,m}w_{A\_C}+C_{n,m}w_{C\_A}\\ B_{n,m}w_{B\_C}+C_{n,m}w_{C\_B}\\ A_{n,m} \end{array} & \begin{array}{c} m\in \left(M_{A}\cap M_{B}\cap M_{C}\right)\\ m\in \left(M_{A}\cap M_{B}\right)\backslash M_{C}\\ m\in \left(M_{A}\cap M_{C}\right)\backslash M_{B}\\ m\in \left(M_{B}\cap M_{C}\right)\backslash M_{A}\\ m\in M_{A}\backslash \left(M_{B}\cup M_{C}\right) \end{array}\\ B_{n,m} & m\in M_{B}\backslash \left(M_{A}\cup M_{C}\right)\\ C_{n,m} & m\in M_{C}\backslash \left(M_{A}\cup M_{B}\right) \end{array}\right..$$

We note that the sum of all given weights ($w_{A}$,$w_{B}$ and $w_{C}$) is 1. For two‐field HLA allele markers present in a proper subset of all reference panels (at least two panels), MultiCook combines the posterior probabilities at the intersection. For those alleles at the intersection, MultiCook rescales the weights to make the sum 1. For example, if an allele is present in two reference panels, the weights of two reference panels are rescaled as $w_{X\_Y}=w_{X}/\left(w_{X}+w_{Y}\right)$, $w_{Y\_X}=w_{Y}/\left(w_{X}+w_{Y}\right)$ and $w_{X\_Y}+w_{Y\_X}=1$ for arbitrary reference panels X and Y. For two‐field HLA alleles in only one reference panel, MultiCook directly adopts the posterior probability from that reference panel as the final combined probability. Equation (2) can be expanded to include additional terms when dealing with more than three reference panels.

**Step 3**. Calling the two‐field HLA alleles.

MultiCook calls the two‐field HLA alleles based on the combined posterior probabilities (Figure 1C). For each individual and HLA gene, MultiCook identifies the most likely pair of alleles using the combined posterior probabilities ($P_{n,m}$), which are obtained from Equation (2). For the nth individual, let $m_{1}$ be the allele with the highest combined posterior probability, followed by the $m_{2}$ with the second‐highest combined posterior probability. Then, MultiCook calls a pair of two‐field HLA alleles as:

(3) $$\mathrm{HLA}\;\text{alleles of}\;\mathrm{nth}\;\text{individual}=\left\{\begin{array}{cc} \left(m_{1},m_{1}\right) & \text{if}\;P_{n,m_{1}}\geq 2P_{n,m_{2}}\\ \left(m_{1},m_{2}\right) & \text{if}\;P_{n,m_{1}}<2P_{n,m_{2}} \end{array}\right..$$

If a pair of two‐field HLA alleles is called ($m_{1},m_{1}$) the genotypes are homozygous. If a pair of two‐field HLA alleles is called ($m_{1},m_{2}$), the genotypes are heterozygous. For example, suppose that an individual has the highest $P_{n,m}$ value of 0.8 for HLA‐A*01:03 and the second‐highest value of 0.2 for HLA‐A*02:02. In this case, MultiCook calls the homozygous genotype (HLA‐A*01:03, HLA‐A*01:03) because 0.8>2×0.2. By contrast, if another individual has the highest $P_{n,m}$ value of 0.8 for HLA‐A*01:03 and the second‐highest value of 0.5 for HLA‐A*02:02, MultiCook calls the heterozygous genotype (HLA‐A*01:03, HLA‐A*02:02) because 0.8<2×0.5.

### 2.2 Merge of HIBAG's Results

MultiCook can merge results from various HLA imputation methods, including CookHLA, the Michigan imputation server and HIBAG. For each two‐field HLA allele, CookHLA and the Michigan imputation server provide probabilities for reference homozygous, heterozygous and alternative homozygous categories, which are used to calculate posterior probabilities in Equation (1). In contrast, HIBAG provides probabilities for allele pairs (genotypes) rather than individual alleles, complicating its incorporation into Equation (1).

To incorporate HIBAG's results, MultiCook applies an alternative approach by transforming HIBAG's genotype probabilities to resemble the posterior probabilities of Equation (1). For each two‐field HLA allele, the probabilities of all genotype pairs containing that allele are summed. However, while CookHLA and the Michigan imputation server tend to output the two largest posterior probabilities as values close to 0.5 for heterozygous cases and 1 for homozygous cases, HIBAG tends to output the two largest summed probabilities close to 1 for both homozygous and heterozygous cases. This discrepancy requires adjustment to ensure consistency across methods for integration. To address this issue, we apply a scaling method: if the highest summed probability for an allele is more than twice that of the second‐highest allele, the genotype is classified as homozygous and all summed probabilities remain unchanged, resulting in a value close to 1. Conversely, if the highest summed probability is less than twice that of the second‐highest allele, the genotype is classified as heterozygous and all summed probabilities are halved to approximate a value close to 0.5 for each allele. These scaled summed probabilities are then treated as HIBAG's posterior probabilities, aligning with CookHLA and the Michigan imputation server.

We compared the histograms of the top two posterior probabilities of HIBAG and the Michigan imputation server using Korean data [19] (Figure S1). The comparison shows that the overall distributions of the top two posterior probabilities from both methods are similar. However, HIBAG's posterior probability distribution is more spread out, with more values concentrated below 0.5, compared to the Michigan imputation server's results.

### 2.3 Imputation Accuracy Calculation

We assessed imputation accuracy as suggested by Cook et al. [8]. For each gene, let $\left(X_{n,1},X_{n,2}\right)$ represent the pair of true two‐field HLA alleles and $\left(Y_{n,1},Y_{n,2}\right)$ represent the pair of predicted two‐field HLA alleles for the nth individual. We measured the imputation accuracy as

(4) $$\begin{array}{c} \text{Accuracy}=\frac{1}{2N}\sum_{n=1}^{N}\max\left(I\left(X_{n,1}=Y_{n,1}\right)+I\left(X_{n,2}=Y_{n,2}\right),I\left(X_{n,1}=Y_{n,2}\right)\right.\\ \;+\left(I,\left.X_{n,2},=,Y_{n,1}\right)\right). \end{array}$$

I is the indicator function that returns 1 if it takes ‘True’ value, otherwise, returns 0. We excluded alleles whose names have been deprecated in the recent version of the IPD‐IMGT/HLA database [20].

### 2.4 HLA Panels

We used several publicly available HLA panels to evaluate imputation methods. We used Korean, HapMap European and PanAsian panels (GRCh37) as target data sets for which we imputed HLA after masking. We used 1KG EUR, 1KG EAS, HAN Chinese and T1DGC panels as references for imputation. For each target data, only SNPs within the MHC region after removing ambiguous ones (AT or GC SNPs) were used for imputation. To prevent overfitting, target samples already present in any of the reference panels were excluded.

#### 2.4.1 Korean Panel

The Korean panel [19] includes *HLA‐A*, *‐B*, *‐C*, *‐DPB1*, *‐DQB1* and *‐DRB1*. HLA was genotyped using the NGS of Roche's GS 454 sequencing at the Institute for Immunology and Infectious Diseases and called with algorithms certified by the American Society for Histocompatibility and Immunogenetics. We downloaded the data from the website described in Kim et al. [19]. The Korean panel consists of 5619 SNPs and 413 individuals.

#### 2.4.2 HapMap European Panel

The HapMap European panel [21] includes *HLA‐A*, *‐B*, *‐C*, *‐DQA1*, *‐DQB1* and *‐DRB1*. HLA was genotyped by PCR‐SSOP‐based protocols. We downloaded the data for 124 individuals from the SNP2HLA website [5], retaining 88 unrelated individuals and excluding 39 samples in the 1KG EUR panel. The final HapMap European panel consists of 4,290 SNPs and 49 individuals.

#### 2.4.3 PanAsian Panel

The PanAsian panel [22] includes *HLA‐A*, *‐B*, *‐C*, *‐DPA1*, *‐DPB1*, *‐DQA1*, *‐DQB1* and *‐DRB1*. HLA was genotyped using a sequence‐based typing (SBT) method with taxonomy‐based sequence analysis. This panel includes Singaporean Chinese, Indian, Malaysian, Japanese and Chinese. We downloaded the data for 530 from the SNP2HLA website [5] and excluded 84 samples in the 1KG EAS panel. The final PanAsian panel used in this study consists of 5848 SNPs and 446 individuals.

#### 2.4.4 1000 Genomes (1KG) Panel

The 1KG panels [23] include *HLA‐A*, *‐B*, *‐C*, *‐DQB1* and *‐DRB1*. HLA was genotyped by using the NGS of PolyPheMe [24]. Among the five super populations of 1KG (AFR; *N* = 540, AMR; *N* = 347, EAS; *N* = 504, EUR; *N* = 503 and SAS; *N* = 486), we used East Asian (EAS) and European (EUR) as reference panels. We downloaded the data from the 1000 Genomes project website [23]. The panels (EAS and EUR) consist of 8016 loci.

#### 2.4.5 HAN Chinese Panel

The HAN Chinese panel [18] includes *HLA‐A*, *‐B*, *‐C*, *‐DPA1*, *‐DPB1*, *‐DQA1*, *‐DQB1*, *‐DRB1*, *‐DRB3*, *‐DRB4* and *‐DRB5*. However, we did not use *HLA‐DRB3*, *‐DRB4* and *‐DRB5* in this study. HLA was genotyped by the targeted NGS sequencing and in silico typing software [25]. We downloaded the data from the website described in Zhou et al. [18]. We removed individuals lacking exactly two appearances of the ‘presence’ in the binary markers. The final HAN Chinese panel used in this study consists of 27,780 loci and 9773 individuals.

#### 2.4.6 T1DGC Panel

The T1DGC panel [26] includes *HLA‐A*, *‐B*, *‐C*, *‐DPA1*, *‐DPB1*, *‐DQA1*, *‐DQB1* and *‐DRB1*. HLA was genotyped by SSOP technology, involving PCR amplification of Exons 2 and 3 in Class I and Exon 2 in Class II [26]. We downloaded the data from the SNP2HLA website [5]. The T1DGC panel used in this study consists of 8961 loci and 5225 individuals.

#### 2.4.7 Asian‐Prefit Panel for HIBAG

The Asian‐prefit reference panel [10] includes *HLA‐A, ‐B, ‐C, ‐DPB1, ‐DQA1 ‐DQB1* and *‐DRB1*. HLA was genotyped using SBT, SSO and SSP. The panel was constructed using HLARES (720 individuals) and HapMap CHB + JPT (90 individuals) data sets. The numbers of SNPs used in this study are 260, 336, 347, 273, 341, 348 and 319 for *HLA‐A, ‐B, ‐C, ‐DPB1, ‐DQA1, ‐DQB1* and *‐DRB1*, respectively.

### 2.5 Competing Methods

#### 2.5.1 CookHLA

CookHLA [8] is an expansion of SNP2HLA [5] and a baseline model of MultiCook. CookHLA and SNP2HLA translate the multiallelic HLA data into binary markers compatible with an SNP imputation tool, Beagle. While SNP2HLA employs Beagle v3, CookHLA employs Beagle (v4 and v5). Additionally, while SNP2HLA positions markers centrally within the HLA gene, CookHLA captures information across each exon, encompassing a broader local context for more precise imputation.

While CookHLA offers compatibility with Beagle versions up to 5 [27], in this study, we employed Beagle version 4 [28] because of the compatibility issues with Beagle version 5: when running imputation on the HapMap European data, Beagle version 5 encountered errors when the sample size fell below 100 individuals in our case.

#### 2.5.2 Michigan Imputation Server

The Michigan imputation server [9] offers a user‐friendly HLA imputation with a multiethnic HLA reference. We specifically employed the two‐field multiethnic HLA reference panel v2, which incorporates data from a diverse population of 20,349 samples, including Japanese and Estonian individuals. Phasing was performed using Eagle v2.4, the default option within the server.

#### 2.5.3 HIBAG

HIBAG [10] is based on attribute bagging. HIBAG provides an R package with internal reference panels. In this study, we used the pre‐built reference panel corresponding to the target population's ethnicity. While we aimed to improve imputation performance by using HIBAG results in MultiCook, our observations indicated less effects from incorporating HIBAG output in most cases. For this reason, HIBAG was excluded from our main benchmarking results. Nevertheless, our MultiCook implementation is equipped with the ability to merge HIBAG results, providing flexibility to the choice of users.

### 2.6 Two‐Fold Cross‐Validation for Reference Panel Selection and Weight Optimisation

**FIGURE 2:**
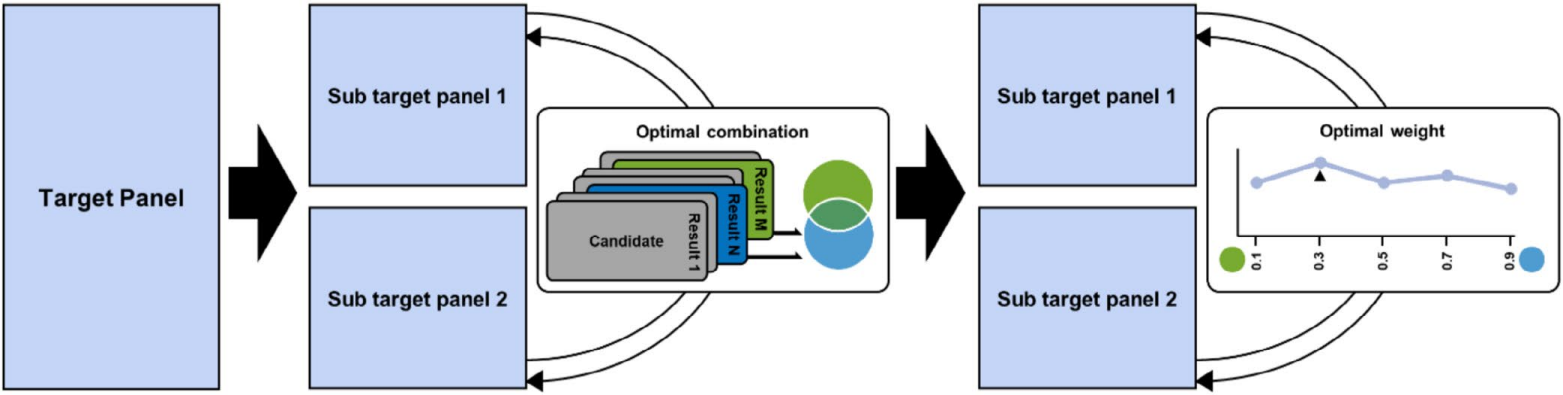
Two‐fold cross‐validation strategy for MultiCook. This figure illustrates the two‐fold cross‐validation strategy used in MultiCook for both reference panel selection and weight optimization. The target data set is split into two subsets, where one subset serves as the validation set to identify the top two reference panels for the other half. After selecting the optimal reference panels, the same cross‐validation strategy is applied to determine the best weights for merging their imputation results.

We performed two‐fold cross‐validation by splitting the target panel into two subsets to prevent overfitting since there was no separate validation HLA panel. We utilised three target data sets: the Korean [23] (*N* = 413), PanAsian [22] (*N* = 446) and HapMap European panel [21] (*N* = 49) target panels in this study. We wanted to merge the results of the top two classical methods with the highest accuracies within MultiCook. To determine the best candidates for merging, we first evaluated each method's accuracy across all available reference panels. However, using the same data for both candidate selection and evaluation can lead to overfitting. To prevent this, we employed a two‐fold cross‐validation strategy for each target data set (Figure 2). The target data set was split into two equal‐sized subsets: one subset served as the validation set to select the two best‐performing reference panels for the other half. The process was repeated by swapping the validation and test sets. The top two selected reference panels remained consistent across both validation directions, confirming the robustness of this approach. Consequently, for the Korean data, we combined CookHLA results based on the HAN Chinese [18] (*N* = 9,773) and the 1KG EAS [23] panel (*N* = 504). For the PanAsian data, we combined CookHLA results based on the T1DGC [26] panel (*N* = 5225) with the Michigan imputation server's results [9]. For the HapMap European data, we combined CookHLA results based on the T1DGC panel with the Michigan imputation server's results.

Once the optimal reference panels were selected, the next step was to determine the optimal weights for merging their imputation results. MultiCook requires weights as hyperparameters to balance contributions from different panels. To find optimal weights, we again employed a two‐fold cross‐validation strategy. Using the previously split subsets, one subset was used as the validation set to tune the weights that maximised imputation accuracy for the test set. This process was repeated by swapping the validation and test sets. The optimal weights remained exactly consistent across both validation directions, given our step size of 0.1. The final optimal weights were assigned as follows: for the Korean data, equal weights (0.5) were assigned to the HAN Chinese panel and the 1KG EAS panel. For the PanAsian data, a weight of 0.6 was assigned to the Michigan imputation server, while 0.4 was assigned to the T1DGC panel. For the HapMap European data, a weight of 0.6 was assigned to the T1DGC panel, while 0.4 was assigned to the Michigan imputation server.

## 3 Results

### 3.1 Accuracy Comparison With Competing Methods

We compared the accuracy of MultiCook with competing methods: CookHLA and the Michigan imputation server. We used three benchmark data sets: the Korean, the PanAsian and the HapMap European target data sets (see Section 2 for details of these panels). Each target data set was imputed using CookHLA or the Michigan imputation server. The final accuracy measurements were computed using two‐field HLA alleles, which were grouped into functionally indistinguishable protein sequences at the P‐group level for evaluation.

**FIGURE 3:**
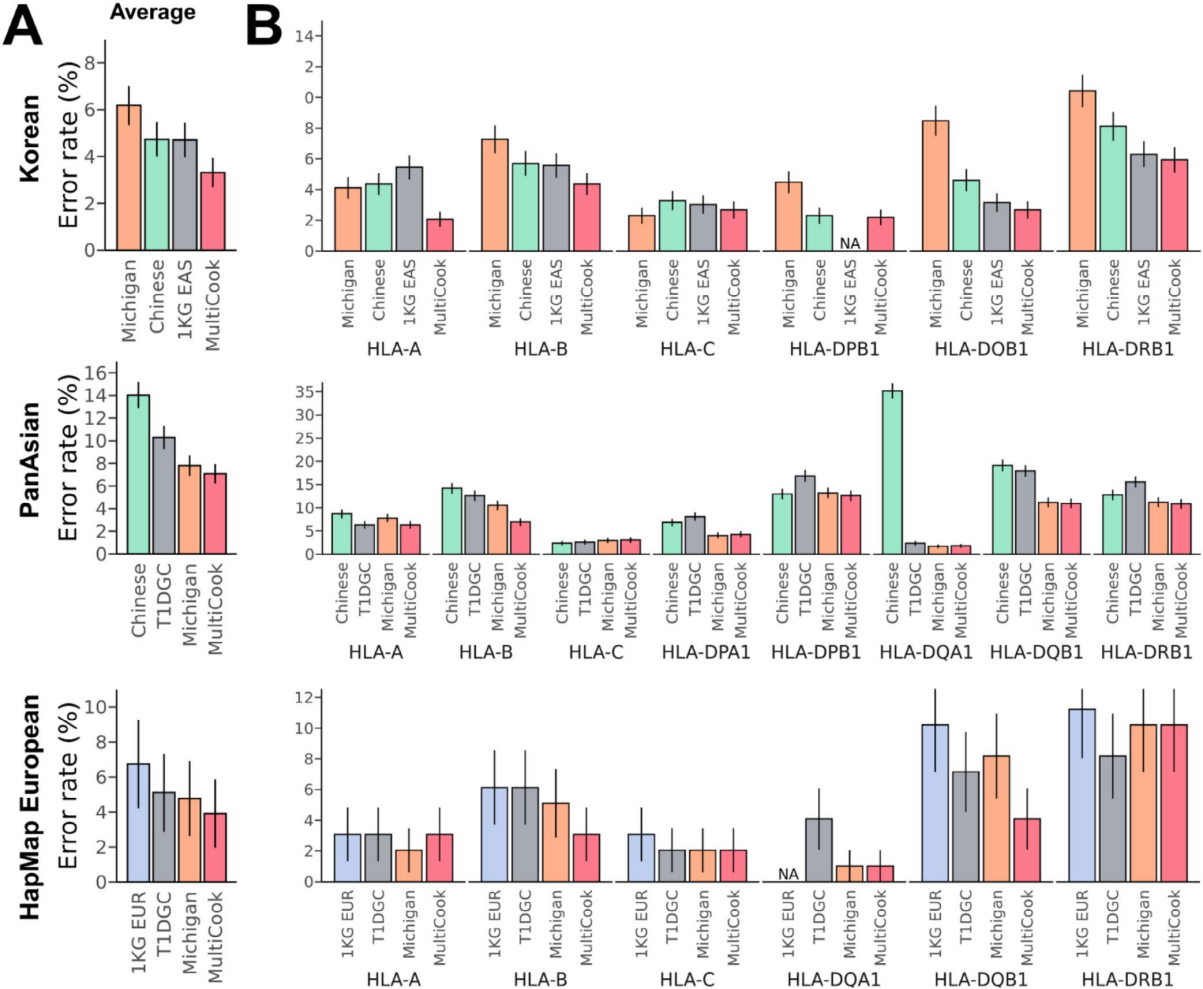
Comparison of imputation error rates. Comparing the error rates of MultiCook to those of single‐panel‐based methods (CookHLA and Michigan imputation server) across the Korean (*N* = 413), HapMap European (*N* = 49) and PanAsian (*N* = 446) data sets. (A) Comparison of average error rates of whole HLA genes. (B) Comparison of error rates per HLA gene.

Our analysis revealed that MultiCook consistently achieved the lowest error rate across the target panels (Figure 3). In imputing the Korean data, MultiCook achieved a lower average error rate (3.31%) compared to other methods' error rates: 4.70% of CookHLA using the 1KG EAS reference panel and 4.72% of CookHLA using the HAN Chinese reference panel. The Michigan server showed a higher error rate of 6.18% compared to both MultiCook and CookHLA. MultiCook's imputation error was about one‐third lower than that of CookHLA based on 1KG EAS, the second‐best performing method and was about a half of that of the Michigan imputation server. This superiority was observed across all HLA types except for *HLA‐C*. Particularly, for *HLA‐A*, MultiCook achieved a remarkable error rate reduction; the error rate was only 2.06% while CookHLA based on the 1KG EAS and HAN Chinese panels showed higher error rates of 5.45% and 4.36%, respectively—more than twice that of MultiCook.

In imputing the PanAsian data, MultiCook achieved a lower average error rate of 7.09% compared to other methods' error rates: 10.28% of CookHLA using the T1DGC panel and 14.02% of CookHLA using the HAN Chinese panel. The Michigan imputation server showed 7.81%. For the PanAsian data set, we observed that using a European reference panel (T1DGC) resulted in a lower error rate than using an Asian panel (HAN Chinese). Among the 8 HLA genes, MultiCook reduced the error rate the most in *HLA‐B*: 6.93% error rate of MultiCook, 12.59% of CookHLA (T1DGC), 14.21% of CookHLA (HAN Chinese) and 10.51% of the Michigan imputation server. Consistent with the previous research [17], we found that a multiethnic‐based model (the Michigan imputation server and MultiCook) performed better than a single‐ethnic‐based model for *HLA‐DPA1* imputation.

In imputing the HapMap European data, MultiCook achieved a lower average error rate (3.92%) compared to other methods' error rates: 5.11% of CookHLA using T1DGC reference panel, 6.74% of CookHLA using 1KG EUR reference panel and 4.77% of the Michigan imputation server. Notably, MultiCook decreased the error rate of *HLA‐DQB1* by a half compared to the Michigan imputation server (4.09% error rate of MultiCook and 8.17% of the Michigan imputation server). In contrast to the imputation of the Korean data, which is underrepresented compared to the European panel, the Michigan server was better than single‐panel CookHLA for imputing the HapMap European data.

MultiCook achieved better stability (more consistent error rates across HLA genes) while the Michigan imputation server's imputation accuracy fluctuated across HLA genes. This variation in accuracy was reflected in the variance of error rates, which was lower for MultiCook (2.33) than for the Michigan imputation server (9.31) in imputing the Korean data. This stable performance across genes highlights MultiCook's robustness as an imputation tool. Similarly, in imputing the PanAsian panel, MultiCook showed greater stability than the Michigan imputation server, with a variance in error rates of 16.04 compared to 19.16 for the Michigan imputation server. In imputing the HapMap European panel, the variance of MultiCook was 11.03 while the variance of the Michigan imputation server was 14.01.

### 3.2 Accuracy Comparison Across Allele Frequencies

We compared the accuracy of MultiCook with other methods across a range of minor allele frequencies (MAFs). Similar to the previous section, we considered imputing HLA for three target data sets: the Korean, PanAsian and HapMap European panels. We calculated allele frequencies based on all three target data sets simultaneously (total *N* = 908). If an allele was present in one or two data sets, the allele frequency was calculated based on the data sets including the allele. Finally, two‐field HLA alleles were grouped into five bins based on their allele frequencies: 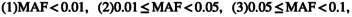 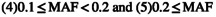. After binning alleles according to the frequencies, we calculated the accuracy as the proportion of correctly imputed alleles in each bin. For this analysis, we used nominal two‐field accuracy: if the digits are equal between true and imputed alleles in two‐field.

**FIGURE 4:**
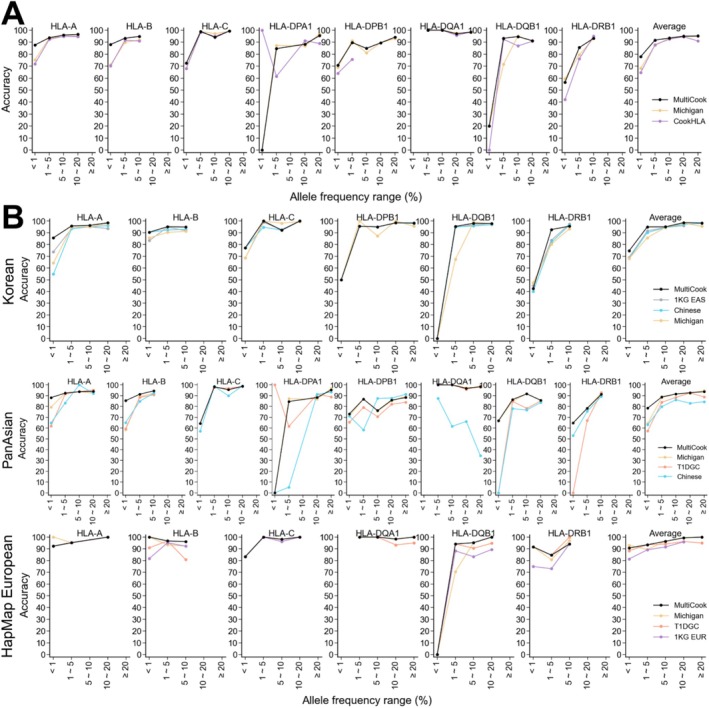
Comparison of imputation accuracies across the minor allele frequency bins. Merging three target data sets (Korean, HapMap European and PanAsian), we calculated minor allele frequency for each two‐field HLA allele. With this frequency, we grouped the alleles into five bins. (A) Average accuracies calculated using three target data sets. (B) Accuracies calculated based on each target data set.

Using all samples in three target panels, we first evaluated the accuracy of MultiCook compared to the Michigan imputation server and CookHLA. (Figure 4A). In this figure, for CookHLA, we used the most accurate reference panel for each target data set to calculate accuracy: the 1KG EAS panel for the Korean data set and the T1DGC panel for both the HapMap European and PanAsian data sets. In most bins, MultiCook achieved better accuracies than other imputation methods. When comparing the average accuracy, MultiCook achieved a higher accuracy of 77.90% compared to 68.21% of the Michigan imputation server and 64.56% of CookHLA for rare alleles with MAF<0.01. When comparing the accuracy across HLA genes, particularly, in *HLA‐A* and *HLA‐B*, MultiCook showed considerably superior accuracy (88.23% and 85.36%, respectively) compared to the Michigan imputation server (79.41% and 58.53%) and CookHLA (71.91% and 70.18%).

We then analysed each target panel separately, for the same analysis of comparing the accuracies across a range of MAFs (Figure 4B). In imputing the Korean target data set, MultiCook resulted in the most superior accuracy on average, followed by CookHLA based on the 1KG EAS, CookHLA based on the Han Chinese panel and the Michigan imputation server. Among the HLA genes, MultiCook achieved notable accuracy improvement, particularly in *HLA‐A* than other methods; in *HLA‐A*, MultiCook showed 85.71% accuracy in rare alleles with MAF<0.01 while CookHLA based on the 1KG EAS and HAN Chinese panel showed 73.80% and 54.76%, respectively. In imputing the PanAsian target data set, MultiCook achieved notable accuracy improvement in *HLA‐A* and *‐B* than other methods. In rare alleles of *HLA‐A*, MultiCook showed 88.23% accuracy, which was notably higher than the second‐best performing method, the Michigan imputation server (79.41%). In rare alleles of *HLA‐B*, MultiCook showed 85.36% accuracy, which was considerably higher than the second‐best performing method, CookHLA based on the HAN Chinese panel (65.04%).

In contrast to imputation of Asians (Korean and PanAsian), in imputing the HapMap European target data set, the Michigan imputation server often achieved better average accuracy than MultiCook for a certain range of rare alleles. For example, for rare alleles with MAF<0.01, the Michigan imputation server showed 93.02% on average while MultiCook showed 90.69%. Except for the rare alleles, though, MultiCook showed comparable or superior average accuracy to the Michigan imputation server. In details, for low‐frequency alleles with 0.01≤MAF<0.05, MultiCook achieved 93.46% on average which is higher than 89.44% of the Michigan imputation server. Recall that for imputing the HapMap Europeans, MultiCook was a combination of CookHLA based on the T1DGC panel and the Michigan imputation server. Thus, merging information of the T1DGC panel with the Michigan imputation server helped overall imputation. But for some rare alleles, merging may not always help as the T1DGC panel is obviously smaller than the Michigan imputation server panel. Note that in Figure 4A, while only the Michigan imputation server seems to accurately impute rare alleles of *HLA‐DPA1* with MAF<0.01, this finding is limited as this bin includes only one allele (HLA‐DPA1*0104), with a total count of 3. In summary, MultiCook was overall superior to other methods in imputing alleles with diverse frequency across all tested target data sets, except for imputing rare alleles (MAF<0.01) of the HapMap Europeans.

### 3.3 Expansion of Allelic Coverage

**FIGURE 5:**
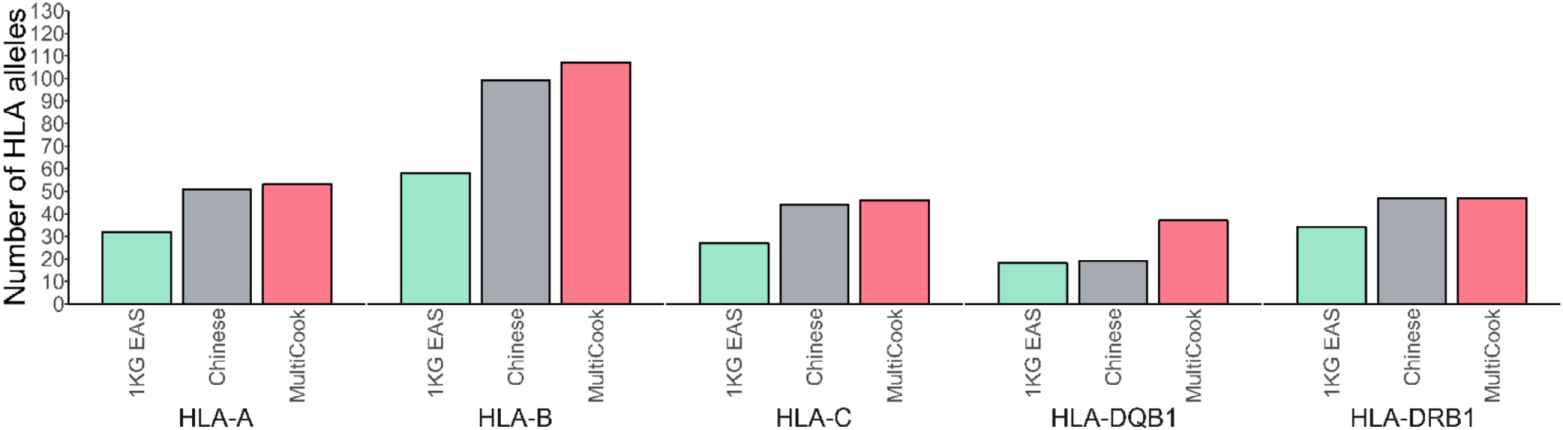
Coverage of two‐field HLA alleles. MultiCook covers more alleles than single‐panel‐based CookHLA as MultiCook is based on the union of multiple allelic sets. In imputing the Korean data, the number of alleles was 290 in MultiCook whereas there were 260 and 169 in the Chinese and 1KG EAS panels, respectively.

By using diverse reference panels at once, MultiCook can cover more alleles compared to single‐panel‐based methods such as CookHLA or HIBAG. Figure 5 illustrates the number of alleles covered by MultiCook and CookHLA for each gene in imputing the Korean data, assuming that MultiCook merges the HAN Chinese and the 1KG EAS panels. MultiCook covers 290 alleles across eight genes, whereas CookHLA based on each of the HAN Chinese and the 1KG EAS panels covers 260 and 169 alleles, respectively. In detail, MultiCook covered 53, 107, 46, 37 and 47 alleles for *HLA‐A*, *‐B*, *‐C*, *‐DQB1* and *‐DRB1*, respectively. On the other hand, for the single‐panel‐based method, CookHLA based on the HAN Chinese panel covered 51, 99, 44, 19 and 47 alleles for *HLA‐A*, *‐B*, *‐C*, *‐DQB1* and *‐DRB1*, respectively. And CookHLA based on the 1KG EAS panel covered 32, 58, 27, 18 and 34 alleles for *HLA‐A*, *‐B*, *‐C*, *‐DQB1* and *‐DRB1*. To assess the representativeness of two‐field HLA alleles in our Korean panel, we conducted a comparative analysis of MAFs between two data sets: our study panel of 413 Koreans and a larger general HLA panel comprising 4128 Koreans [29]. This comparison focused on alleles from the *HLA‐A*, *HLA‐B* and *HLA‐DRB1* loci. We found a high correlation between the two panels, with a Pearson correlation coefficient of 0.98. The results are summarised in Figure S2 and Table S1.

### 3.4 Impact of Weights on Imputation Accuracy

**FIGURE 6:**
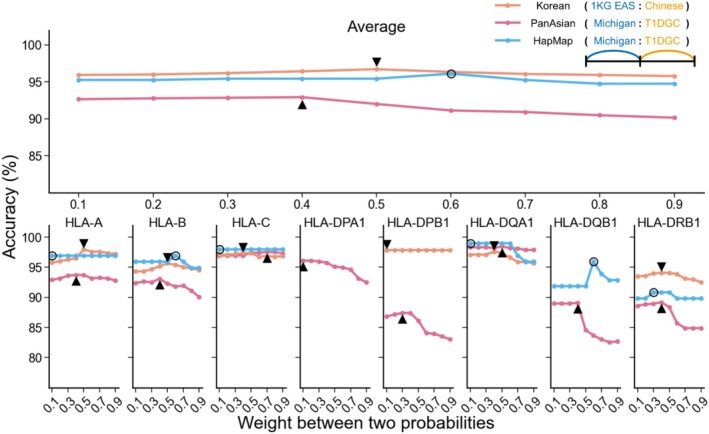
Merging weights affecting the imputation accuracy. In imputing the Korean, PanAsian and HapMap European data sets, we varied the weights of linear combination to investigate the optimal weights between reference panels. The weights on the *x* axis are given to the HAN Chinese relative to 1KG EAS for the Korean data set and T1DGC relative to the Michigan server for both the PanAsian and HapMap European data sets. The triangle and circle icons show the points with the best accuracy.

We investigated the impact of the merging weights in MultiCook on imputation accuracy (Figure 6). When merging imputation results from runs based on independent reference panels, MultiCook can incorporate weights based on the informativeness of each panel (see Section 2). A simple strategy would be to apply equal weights, but applying unequal weights can often make differences. In this analysis, for each of the target data sets (Korean, PanAsian and HapMap European), we varied the weights from 0.1 to 0.9. When imputing the Korean data, assigning equal weight (0.5) to the HAN Chinese and 1KG EAS panels yielded the highest average accuracy (96.69%). This finding reflects the similar accuracies of CookHLA based on the HAN Chinese and 1KG EAS panels in Figure 3. We further assessed the impact of weights for imputing the PanAsian data between the Michigan imputation server and CookHLA based on the T1DGC panel. Assigning a greater weight (0.6) to the Michigan imputation server compared to the T1DGC panel (0.4) resulted in the highest average accuracy (92.91%). This finding reflects the superior performance of the Michigan imputation server compared to CookHLA based on the T1DGC. In contrast, assigning a greater weight (0.6) to the T1DGC panel compared to the Michigan imputation server (0.4) resulted in the highest average accuracy (96.06%) for imputing HapMap European data.

We also investigated the variability of weight impact across HLA genes in MultiCook. In imputing the Korean and PanAsian data sets, MultiCook showed the greatest accuracy improvement in *HLA‐B*, demonstrably shaping the peak accuracy at 0.5 in the graph. The mountain shape shows that to improve accuracy in *HLA‐B*, merging the information from both panels was effective. In imputing *HLA‐DPB1*, *‐DQB1* and *‐DRB1* alleles of the PanAsian data, MultiCook showed a dramatic decrease in accuracy when the weight assigned to the T1DGC panel exceeded 0.4. This shows that for the Michigan imputation server to contribute to the results, a weight of at least 0.6 was needed. In imputing *HLA‐DQB1* alleles of the HapMap European data, MultiCook gained the top accuracy improvement when the Michigan imputation server was weighted exactly at 0.6, suggesting that there may indeed exist a certain point in weights that the effectiveness of merging can be maximised.

### 3.5 HLA Panel's Impact on HIBAG Integration

We evaluated the effectiveness of incorporating HIBAG's imputation results. Since HIBAG did not rank among the top two performing methods, its results were not included in Figure 3. For this evaluation, we used the Korean panel as our target data. For this target panel, we first compared the individual imputation results of HIBAG (Asian‐prefit) with those of EAS‐based CookHLA and the Michigan imputation server (Figure S3). Note that Asian‐prefit refers to running HIBAG with pre‐fitted parameters provided by the HIBAG package for the Asian population [10]. Among these three methods, EAS‐based CookHLA achieved the lowest average error rate at 4.47%. Michigan imputation followed with an error rate of 6.17%, while HIBAG had the highest error rate at 6.98%.

Subsequently, we examined the impact of merging HIBAG's results with the other two. Merging HIBAG's results with Michigan imputation server's results reduced the average error rate to 5.16% from Michigan's original 6.17%. Conversely, merging HIBAG with EAS‐based CookHLA increased the error rate to 4.76% compared to CookHLA alone at 4.47%. These findings suggest that incorporating HIBAG can be beneficial if the accuracy of HIBAG is similar to the panel to be merged. By contrast, in the case that the accuracy of the panel to be merged was superior to HIBAG's model, incorporating HIBAG did not help.

### 3.6 Evaluation of Weighting Strategies in MultiCook

To further investigate whether optimal weights are related to the sample size of the reference panel, we conducted additional research. In imputing the Korean target panel, we explored three weighting schemes in MultiCook: proportional to sample size, square root of sample size and logarithm of sample size (Figure S4). However, none of these weighting schemes achieved accuracy surpassing the case with equal weights. Therefore, we suggest that the users begin with equal weights if reference panels are expected to provide similar single‐panel performance. If single‐panel accuracy can be approximated a priori, as shown in the weight variation (0.1–0.9) explored in Figure 6, assigning greater weight to a reference panel with notably superior accuracy could lead to improved imputation results. However, this requires knowing the accuracy for the target sample a priori, which is often impractical. Therefore, without prior knowledge of each method's performance, if a user's target ethnicity to impute matches one of the available reference panels, assigning a greater weight to that panel is recommended. Otherwise, using equal weights across all reference panels remains the most practical approach. Further investigation is necessary to determine the most effective method for assigning optimal weights to each reference panel.

## 4 Discussion

We have developed an HLA imputation method, MultiCook, which allows us to simultaneously utilise multiple reference panels and tools. MultiCook effectively improves imputation accuracy by combining information from multiple reference panels. This approach can improve both the reference panel size and the diversity within the reference panel. First, using multiple panels can be thought of as increasing the panel size, which can enhance the imputation accuracy [30]. For example, if the existing ethnicity‐matched panel lacks a sufficient sample size, using multiple reference panels may help. Second, for samples from diverse ethnic backgrounds, diverse HLA reference panels are often required. For example, if there is no single reference panel that matches the target's ethnicity, using diverse reference panels may help.

MultiCook is versatile enough to incorporate the Michigan imputation server's output, but whether using the Michigan imputation server helps varied by situations. In real data benchmarking, we observed that the effectiveness of the use of the Michigan imputation server depended on the relative performance of the Michigan imputation server compared to the single‐panel CookHLA. In the case that the accuracy of the Michigan imputation server is lower than CookHLA's accuracy, combining the results of individual CookHLA results without the Michigan imputation server led to notable improvement. By contrast, in the case that the Michigan imputation server outperformed CookHLA, combining the results of the Michigan imputation server with those of CookHLA increased the imputation accuracy. Regarding the Michigan imputation server, this can be thought of as MultiCook fine‐tuning the results of the Michigan imputation server, increasing accuracy and robustness by utilising external reference panels of CookHLA.

MultiCook can merge results from various imputation methods like CookHLA, the Michigan imputation server and even HIBAG. Merging HIBAG's results based on the pre‐fitted reference panel did not result in the best results. While the merger with HIBAG's results based on a pre‐fitted reference panel decreased accuracy, using an external reference panel might improve future merger. Furthermore, MultiCook's compatibility extends beyond these three HLA imputation tools, as MultiCook can handle results from any future tools as long as their outputs can be interpreted as posterior probabilities.

During imputation benchmarking, we observed some issues regarding performance decrement due to MAF difference of HLA alleles between reference and target panel. For example, in imputing the Korean data, MultiCook utilised the 1KG EAS and the HAN Chinese reference panels (Figure 4B). For *HLA‐C* alleles within the bin of 0.05≤MAF<0.1, MultiCook showed lower accuracy (92.21%) compared to the Michigan imputation server (98.15%). This difference is attributed to HLA‐C*07:01 in the bin. Among 22 Koreans who were heterozygous for HLA‐C*07:01, MultiCook misidentified 21 of these 22 samples as HLA‐C*07:06 which is absent in the Korean data. And this misclassification led to the accuracy decrement. This coincides with the notably lower MAF of HLA‐C*07:01 in both reference panels (0.00547 and 0.00099 for the 1KG EAS and Han Chinese panels) compared to the MAF of the Korean data (0.02663). The observed difference of MAF for HLA‐C*07:01 between the Korean population (target data) and reference populations (reference data) aligns with findings reported in other studies, which can be checked on the official site of Allele Frequency Net Database (https://www.allelefrequencies.net/hla6008a.asp?hla_allele=C*07:01) [31]. A possible misidentification of HLA‐C*07:01 as HLA‐C*07:06 likely stems from the shared nucleotide sequence in exons 2 and 3, which is reported by Delfino et al. [32].

We further analysed the impact of HLA typing methods on the accuracy. We used three types of HLA data sets (NGS, SBT and SSOP). For reference panels, the Michigan imputation server's multiethnicity panels, the HAN Chinese panel and the 1KG panels are based on NGS for HLA typing while the T1DGC panel is based on SSOP. For target panels, the Korean panel is based on NGS, the HapMap European panel is based on SSOP and the PanAsian panel is based on SBT. First, HLA typing methods seem not to affect the imputation, measuring the accuracy based on two fields of two‐field HLA alleles. In imputing the PanAsian and HapMap European data, comparing the single‐panel‐based methods, the imputation using the T1DGC based on SSOP achieved better accuracy than the Chinese reference panel and the 1KG European panels based on NGS, respectively. Second, the consistency of HLA typing methods among the merged reference panels seems to affect the imputation. In imputing the Korean panel, MultiCook merged the results of the HAN Chinese and the 1KG EAS panels, both based on NGS, which showed the most lowest error rate than imputing the PanAsian and HapMap European panels which are based on the merge of NGS (Michigan imputation server) and SSOP (T1DGC). However, these merges may be less effective than the merge for the Korean panel, as they involve multiethnic panels and a single reference panel. Utilising a limited number of HLA reference panels in this research, further research is needed for the effect of HLA typing methods.

For a better research of HLA imputation, international collaboration of researchers is required for HLA imputation research [17, 33]. First, the performance of MultiCook can be enhanced by collaboration. By not only building a large sized reference panel but also merging the imputation results of diverse reference panels corresponding to the ethnicity of the target data set, we can improve the imputation performance. In the case that researchers cannot provide their own HLA reference panels, MultiCook is useful in that they just need to run an imputation using their own HLA reference panels and provide the results for a merge without exposing the reference panels. Also, further research, such as a research of HLA typing impact on imputation, can be done by international collaboration. Second, higher resolution imputation in MultiCook can be done by collaboration. Even though we utilised two‐field alleles which define a single protein sequence [34], we anticipate that more HLA reference with two‐field alleles or higher resolution alleles would be available for HLA imputation through international collaboration, given that NGS‐based typing with higher resolution alleles becomes more prevalent. Last, the comprehensive validation set for the optimal combination of reference panels and weights for them is needed for MultiCook. As the HLA panels are limited, there is a constraint for the validation data set. To find the optimal combination of reference panels and weights, we adopted a two‐fold cross validation approach in which one subset served as the validation set for the optimal combination and weights of another subset. If we gain access to more diverse HLA panels by collaborating with international researchers, we can use more diverse HLA panels, some of them as reference panels, some of them as target panels and some of them as validation panels. Without additional validation data sets, we recommend using the optimised weights from this study, which are specific to European and Asian populations.

The growing recognition of HLA's role in infectious and autoimmune diseases coincides with the declining cost of HLA sequencing, leading to the creation and release of more diverse HLA reference panels. Additionally, the affordability of genome‐wide association study chips is enabling not only large‐scale studies but also individual genotyping in smaller cohorts. Consequently, the demand for accurate HLA imputation methods is growing due to both the availability of diverse HLA reference panels and the increased popularity of SNP genotyping. However, most existing HLA imputation tools rely on a single reference panel with limited diversity and sample size, hindering their ability to address the high polymorphism of the MHC region with extensive genomic variation and length differences [35]. To overcome this limitation, we developed MultiCook, a method that leverages multiple panels for more accurate HLA imputation. We anticipate MultiCook to be a valuable asset for HLA researchers, particularly, those studying underrepresented populations.

## Author Contributions

Buhm Han, Hakin Kim and Hyunjoon Lim designed the study. Hakin Kim and Hyunjoon Lim performed the study. Hakin Kim analysed the data. Hakin Kim implemented a local web‐based platform. Buhm Han and Hakin Kim wrote the manuscript. Buhm Han reviewed the manuscript.

## Conflicts of Interest

Buhm Han is the CEO of SpintoAI Inc.

## Supporting information


**Data S1.** Supporting Information.

**Figure S1.** Comparison of the top two posterior probability distributions of HLA alleles between HIBAG and the Michigan imputation server using the Korean target panel. The distributions are similar, though HIBAG’s posterior probability distribution is slightly more spread out. Michigan imputation server computes posterior probabilities using its multiethnicity panels, whereas HIBAG estimates them using a pre‐fitted Asian panel provided by the package before integrating them in MultiCook.

**Figure S2.** Comparison of minor allele frequencies (MAFs) between our Korean panel (*n* = 413) and a general Korean HLA panel (*n* = 4,128) for *HLA‐A*, *HLA‐B* and *HLA‐DRB1* loci. The high correlation (Pearson’s correlation coefficient, *r* = 0.98) indicates that our study panel is representative of the broader Korean population in terms of two‐field HLA allele distributions.

**Figure S3.** Error rates of MultiCook, which integrates HIBAG with other imputation methods, for HLA imputation in the Korean panel. The figure compares the individual performances of HIBAG (Asian‐prefit), Michigan imputation server and EAS‐based CookHLA with their combined results. The combination of HIBAG and Michigan, which showed similar performance individually, led to a reduced error rate compared to using either method alone.

**Figure S4.** Error rates of MultiCook using different weighting schemes based on sample size for imputing the Korean target panel. Weighting proportional to sample size, square root of sample size and logarithm of sample size did not show improvement over equal weighting.

**Table S1.** Minor allele frequencies (MAFs) of *HLA‐A*, *HLA‐B* and *HLA‐DRB1* alleles in our Korean panel (*n* = 413) and a general Korean HLA panel (*n* = 4,128).

## Data Availability

We used data derived from public domain resources: Korean panel, HapMap European panel, PanAsian panel, 1000 Genomes panel, HAN Chinese panel, and T1DGC panel. Details of each panel are described in the Methods and Materials section, with a summary provided in Table 1.
