# Supplementary material for: MultiCook: A Tool That Improves Accuracy of HLA Imputation by Combining Probabilities From Multiple Reference Panels and Methods
Source: HLA. 2025 May 6;105(5):e70153. doi: 10.1111/tan.70153 (PMC12054343; doi:10.1111/tan.70153)
Supplement: Supplementary file 1 — Data S1. Supporting Information. [file TAN-105-e70153-s004.docx]

Manual for web-based MultiCook

We provide web based imputation, based on python Flask to ease the use of MultiCook for users.

MultiCook can be used in three scenarios:

(1) accessing and running the application on a **Linux server** from a local computer,

(2) running the application directly on a **MacBook** or a **Linux machine**, and

(3) running **only the Merge step (Step 2)** on a **Windows** using pre-existing results.

**On Windows, go directly to step 2 for a merge because CookHLA and HIBAG are not supported on Windows**

For the first scenario (1), connect to the server with
ssh -p [port number] -L localhost:5000:127.0.0.1:5000 [server user ID]@[server IP address]

on the local server’s CMD.

And activate anaconda environment and run Flask on ./MultiCook including app.py with

conda activate MultiCook

flask run.

For the other scenaros, (2) and (3), just activate anaconda environment and run Flask on ./MultiCook with

conda activate MultiCook

flask run.

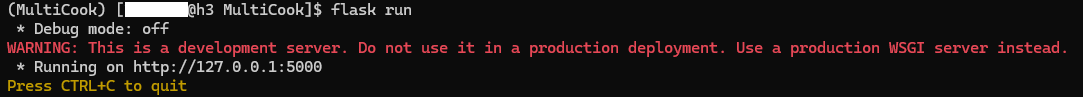


Then copy ‘http://127.0.0.1:5000’ and paste on the browser, which leads to the following image.

The first step is for running single-reference-panel based imputatoin methods (HIBAG or CookHLA).
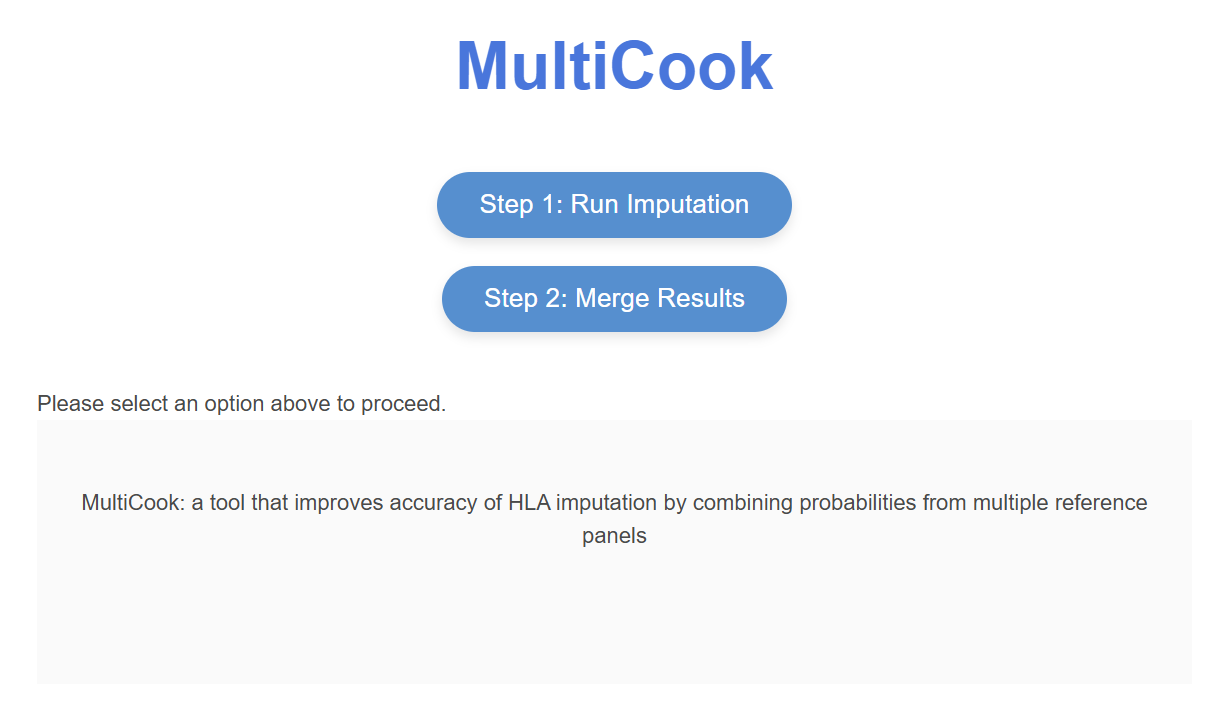

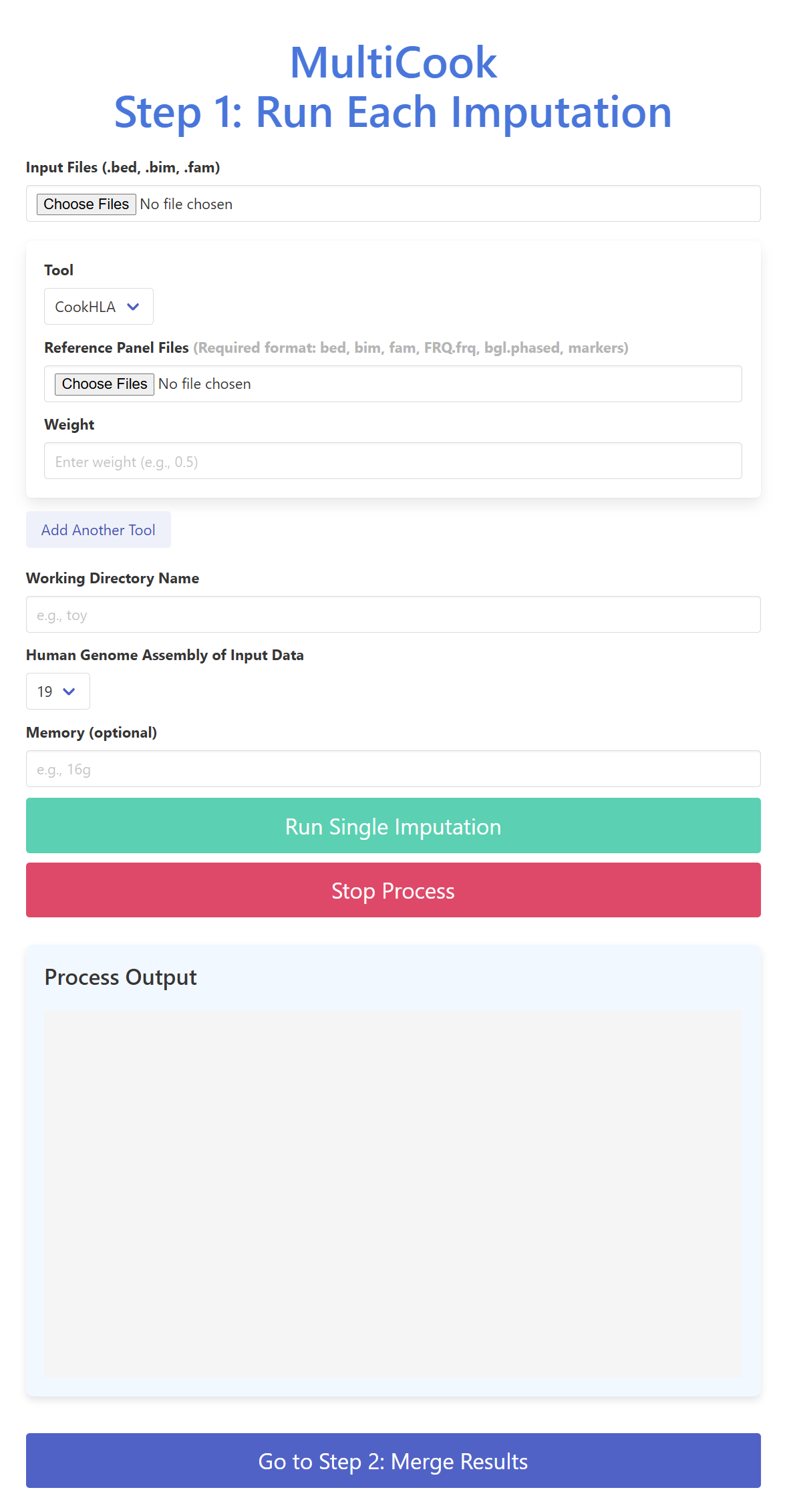


For input files, plink bfiles (bed, bim and fam) are required.


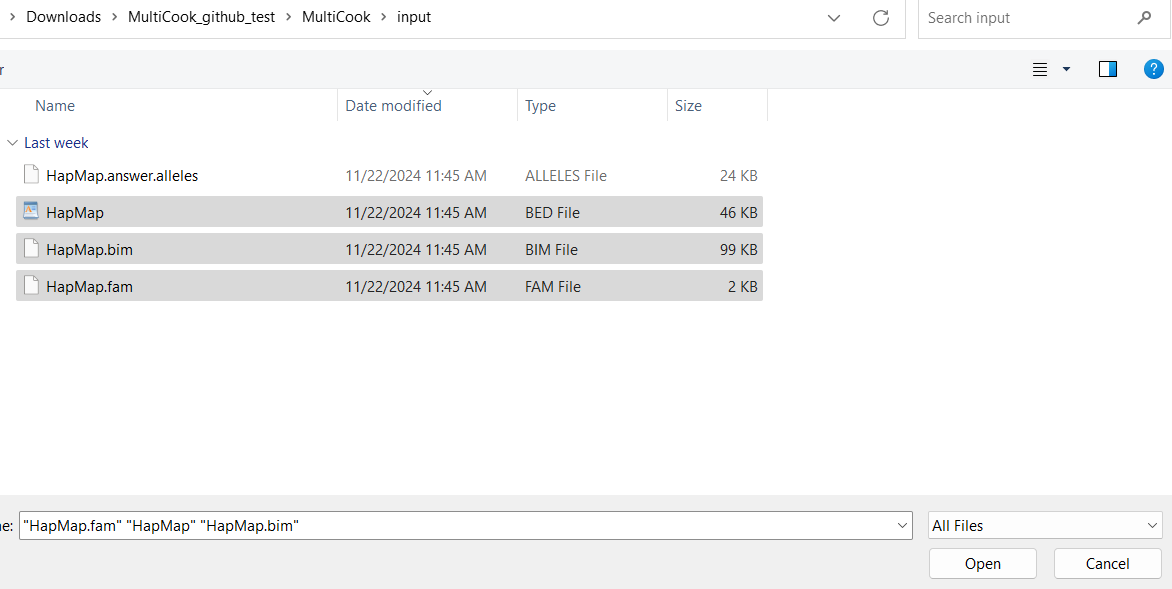


For CookHLA, 6 files (bed, bim, fam, FRQ.frq, bgl.phased and markers) have to be uploaded (selected) simultaneously as a reference panel.


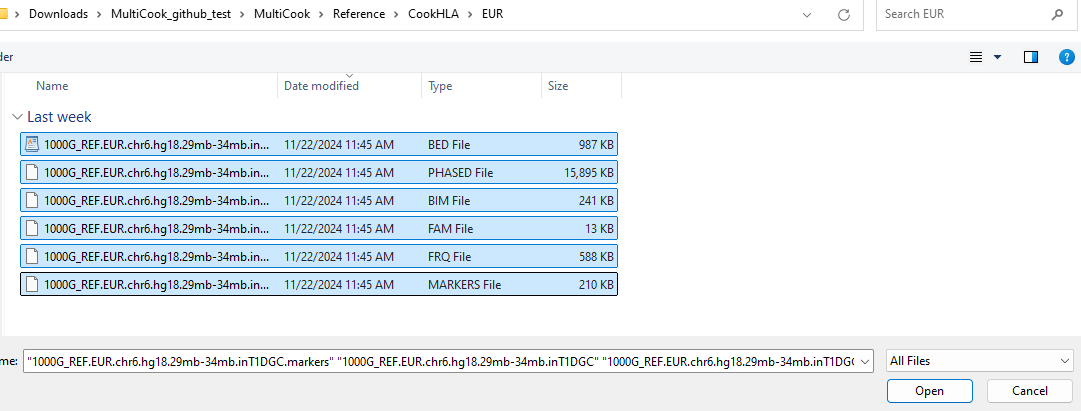


For HIBAG, one file (.RData) has to be uploaded (selected).


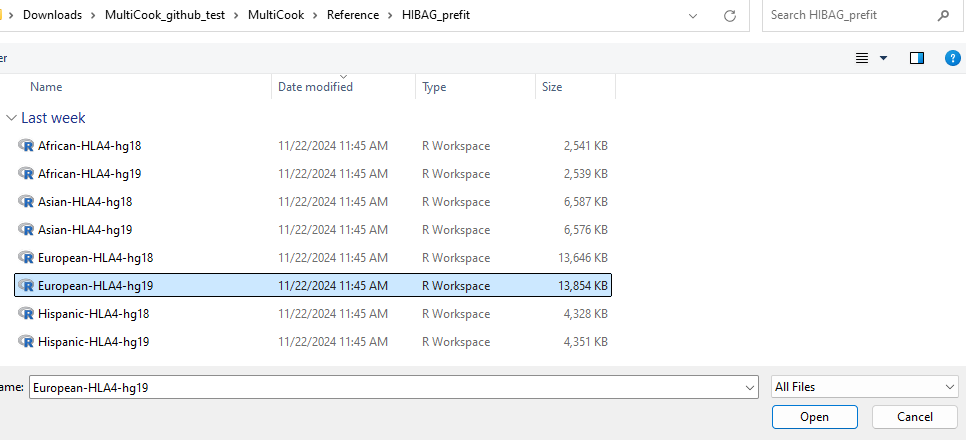


Those uploaded files are automatically saved on ./MultiCook/uploads/{working directory name}.

The memory typed is used for Beagle the engine of CookHLA.

After filling all the required formats of the first web page, click the Run Single Imputation button, then the imputation process would start, each process would be done sequentially


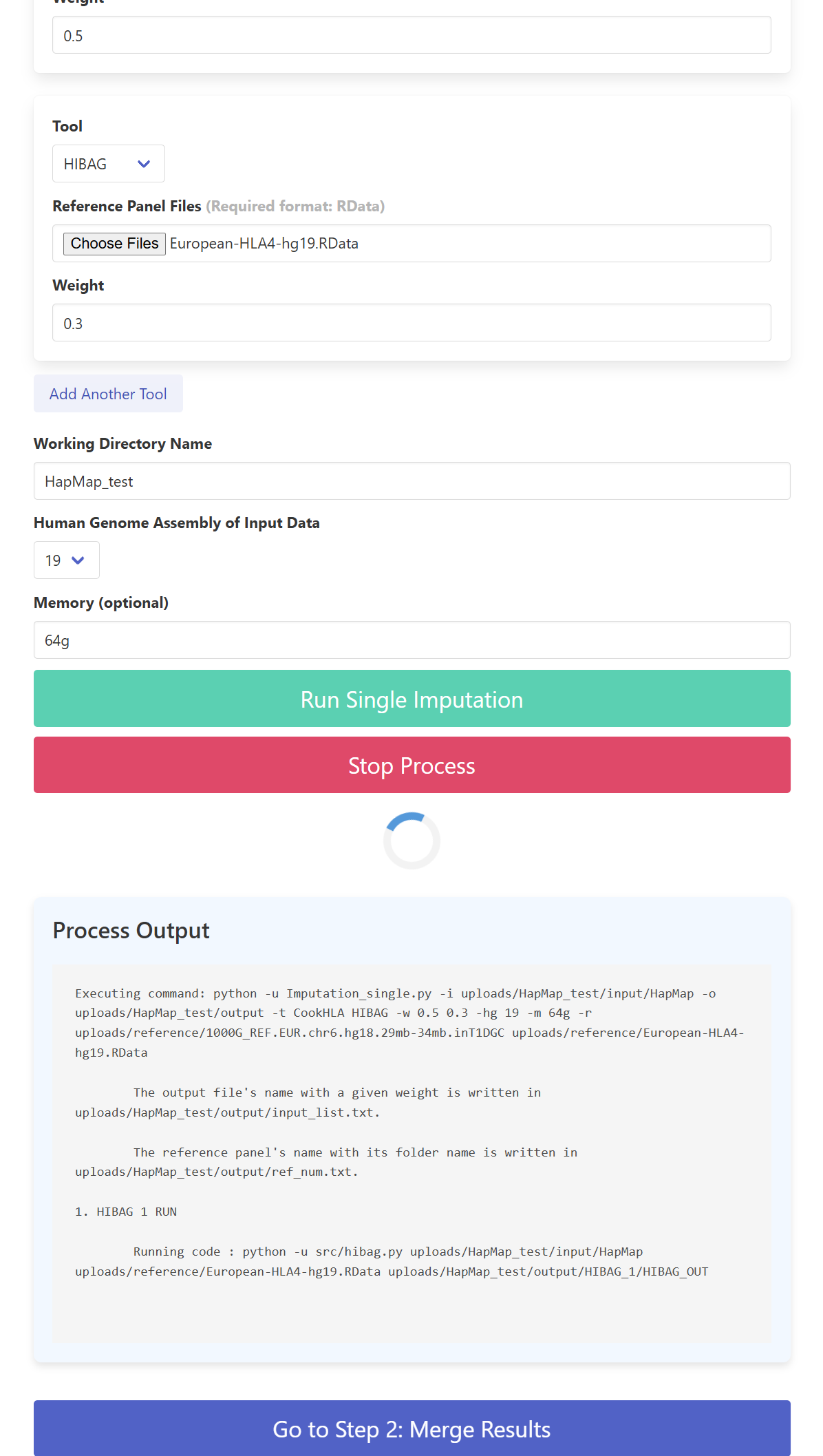


After finishing the single-reference-based imputation, Move to next page for merge.

Note that, if you already ran the imputation on step 1, the results would be already uploaded for a merge. In this case just go to step 2 and click Merge Results button after typing the same working directory name from step 1.

To integrate additional outputs, such as those from the Michigan Imputation Server or other collaborations, upload the corresponding results on this page.


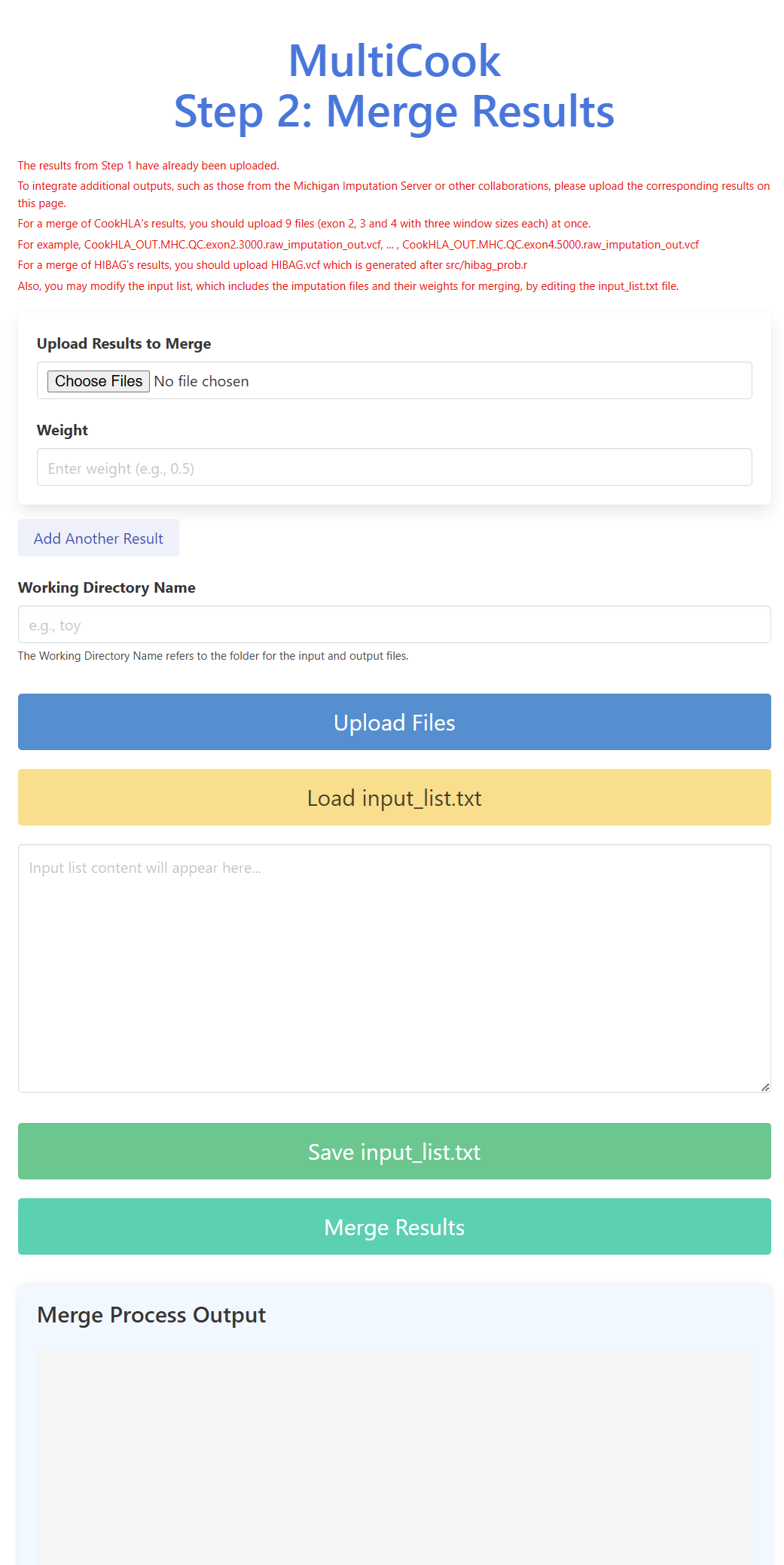


For a merge of Michigan imputation server’s results in vcf.gz format, upload (select) the file and type a weight for it. Usually, chr6.dose.vcf.gz is generated after Michigan imputation.

You should type the working directory name again.

The imputation result files to be merged and their weight is in input_list.txt, which can be edited in real time by Load input_list.txt and Save input_list.txt

The text-edit box must not include extra lines.

After editing the input_list.txt, you must click Save input_list.txt button for an update.


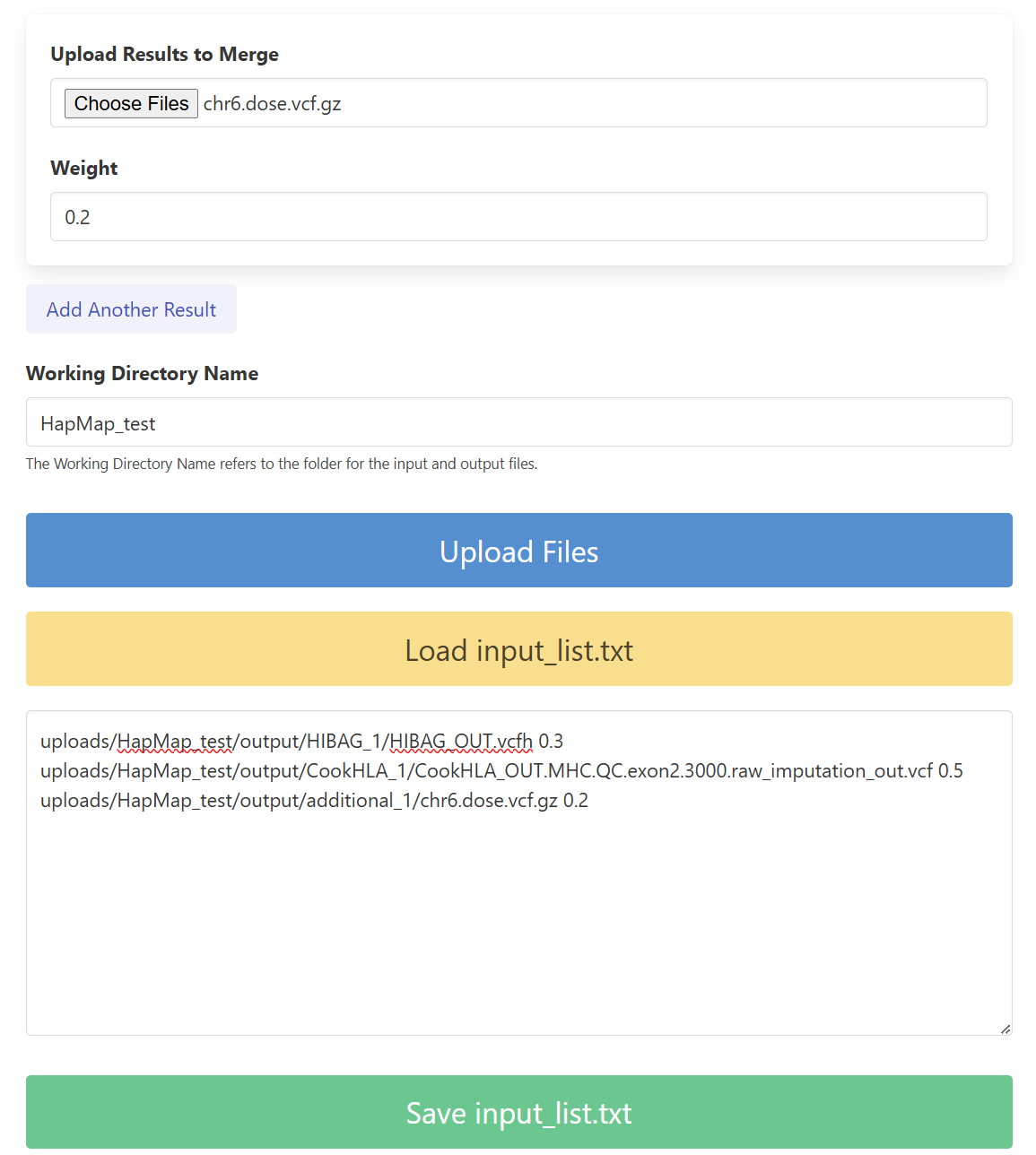


For an additional merge of CookHLA's results, you should upload 9 files (exon 2, 3 and 4 with three window sizes each) at once (CookHLA_OUT.MHC.QC.exon2.3000.raw_imputation_out.vcf, ... , CookHLA_OUT.MHC.QC.exon4.5000.raw_imputation_out.vcf)

For an additional merge of HIBAG's results, you should upload HIBAG.vcf which is generated after executing src/hibag_prob.r which calculates the probabilities required for MultiCook.

After uploads all the results from diverse methods, click Merge Results.


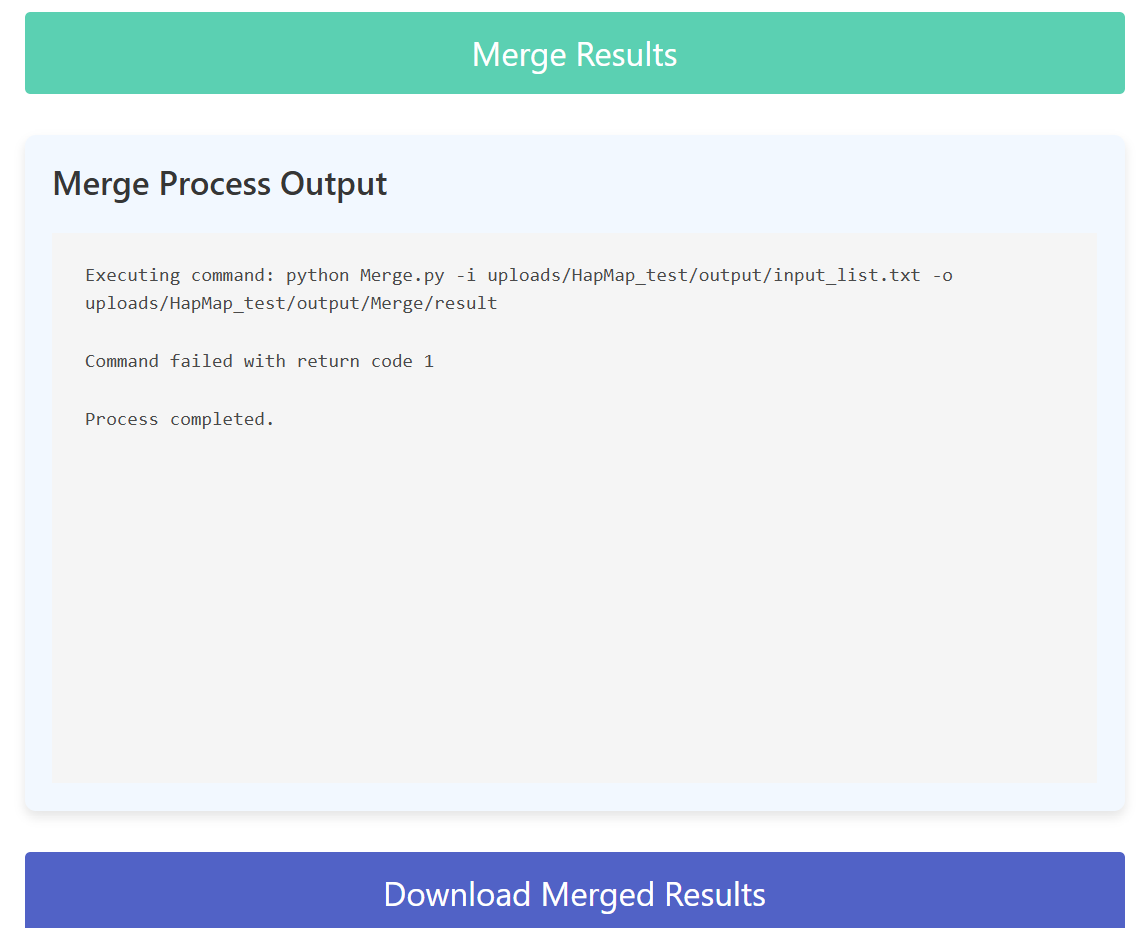


After finishing the merge process, click Download Merged Results, then result.all.alleles file would be downloaded.
