# Supplementary figures and images for: MultiCook: A Tool That Improves Accuracy of HLA Imputation by Combining Probabilities From Multiple Reference Panels and Methods
Source: HLA. 2025 May 6;105(5):e70153. doi: 10.1111/tan.70153 (PMC12054343; doi:10.1111/tan.70153)

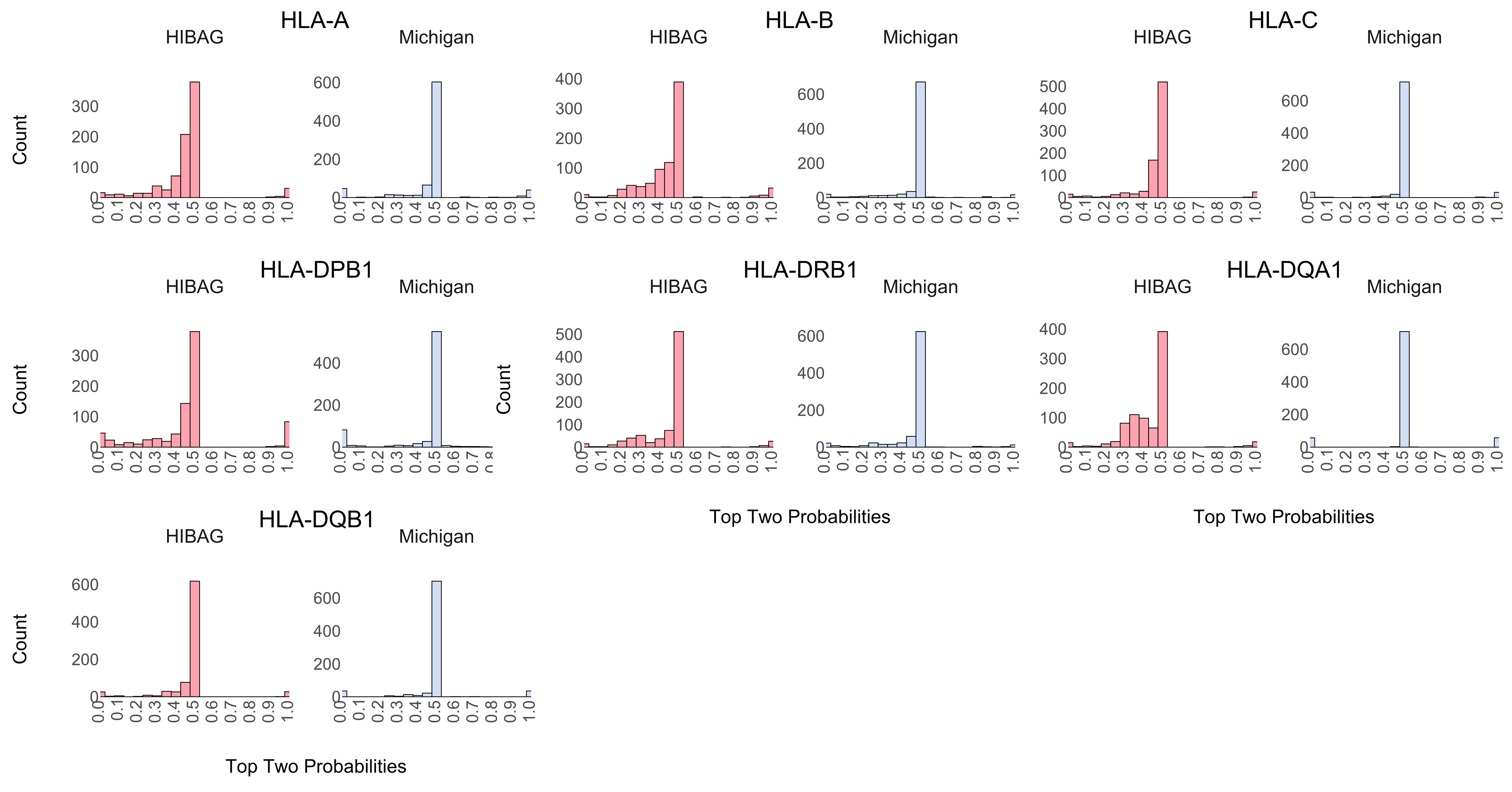

Supplement: Supplementary file 2 — Figure S1. Comparison of the top two posterior probability distributions of HLA alleles between HIBAG and the Michigan imputation server using the Korean target panel. The distributions are similar, though HIBAG’s posterior probability distribution is slightly more spread out. Michigan imputation server computes posterior probabilities using its multiethnicity panels, whereas HIBAG estimates them using a pre‐fitted Asian panel provided by the package before integrating them in MultiCook. [file TAN-105-e70153-s003.png]

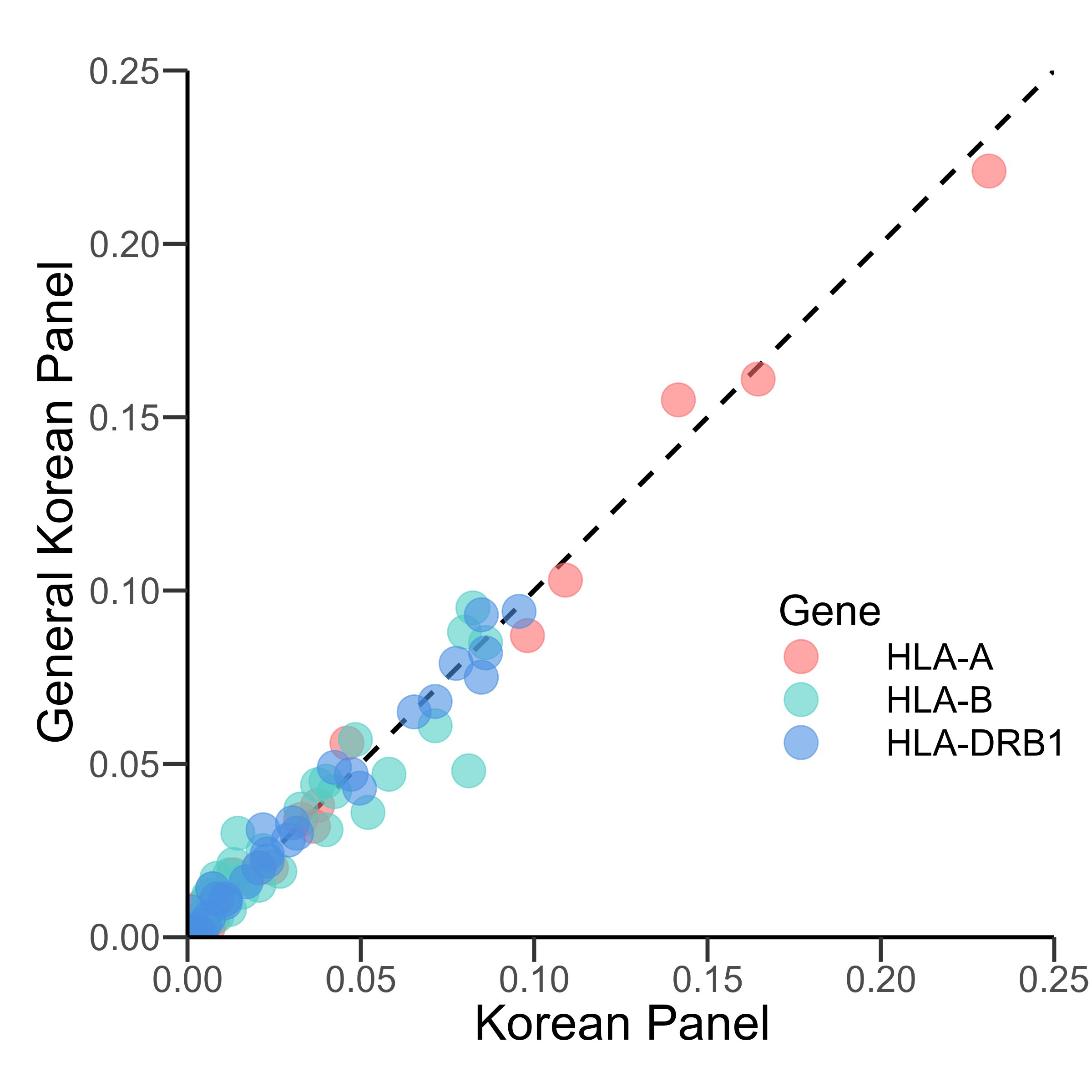

Supplement: Supplementary file 3 — Figure S2. Comparison of minor allele frequencies (MAFs) between our Korean panel (n = 413) and a general Korean HLA panel (n = 4,128) for HLA‐A, HLA‐B and HLA‐DRB1 loci. The high correlation (Pearson’s correlation coefficient, r = 0.98) indicates that our study panel is representative of the broader Korean population in terms of two‐field HLA allele distributions. [file TAN-105-e70153-s002.png]

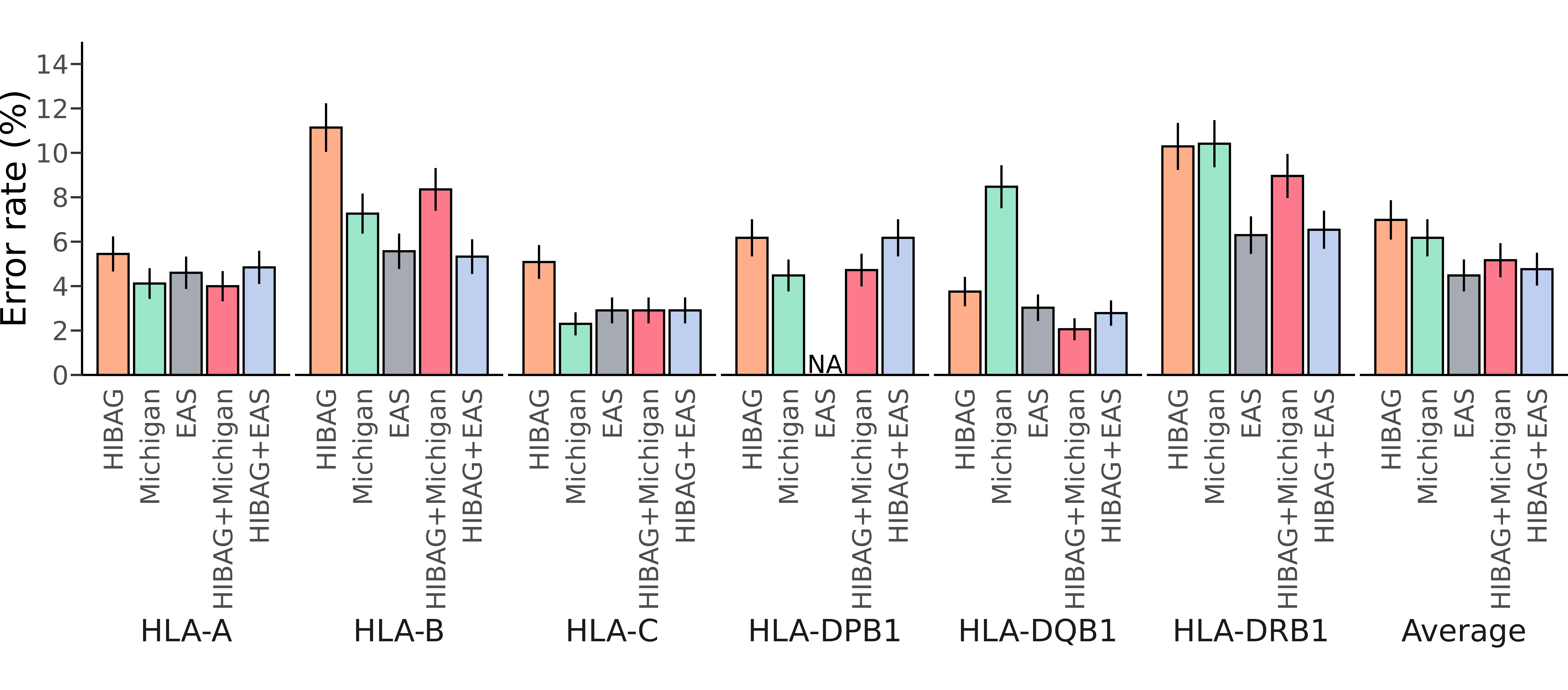

Supplement: Supplementary file 4 — Figure S3. Error rates of MultiCook, which integrates HIBAG with other imputation methods, for HLA imputation in the Korean panel. The figure compares the individual performances of HIBAG (Asian‐prefit), Michigan imputation server and EAS‐based CookHLA with their combined results. The combination of HIBAG and Michigan, which showed similar performance individually, led to a reduced error rate compared to using either method alone. [file TAN-105-e70153-s005.png]

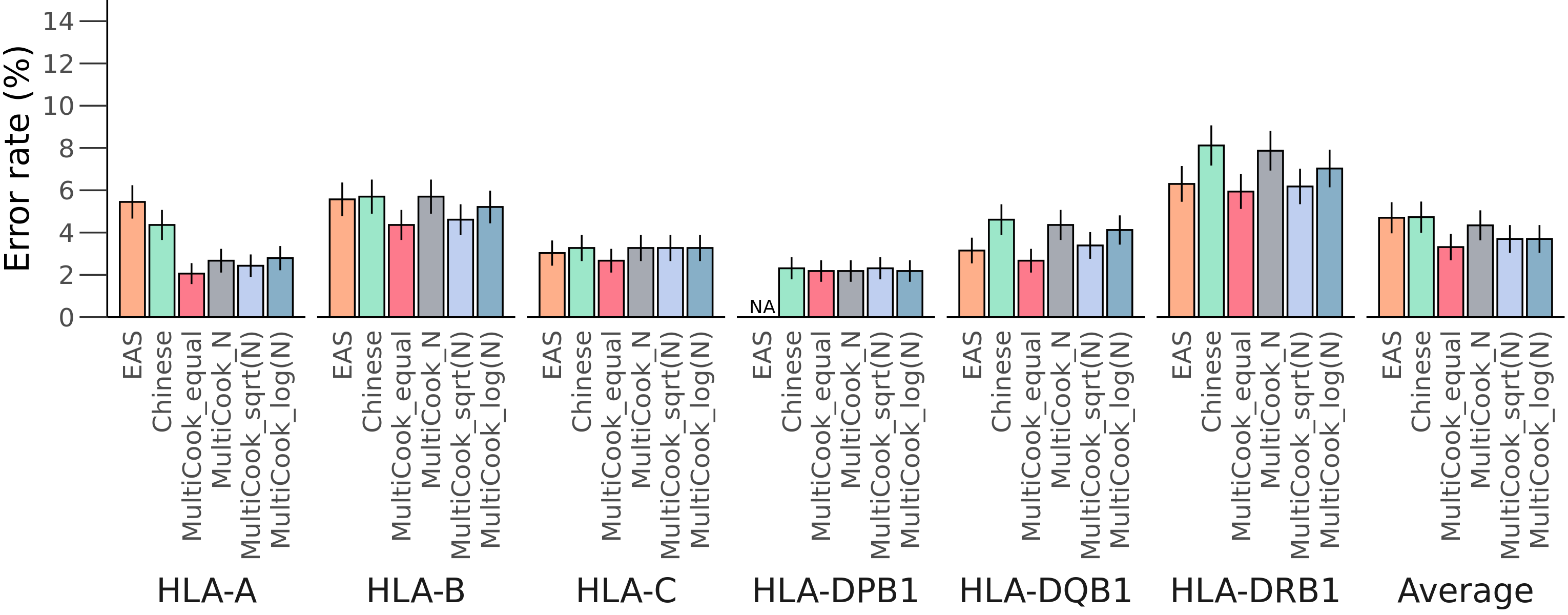

Supplement: Supplementary file 5 — Figure S4. Error rates of MultiCook using different weighting schemes based on sample size for imputing the Korean target panel. Weighting proportional to sample size, square root of sample size and logarithm of sample size did not show improvement over equal weighting. [file TAN-105-e70153-s001.png]
